# Use of classical bird census transects as spatial replicates for hierarchical modeling of an avian community

**DOI:** 10.1002/ece3.4829

**Published:** 2019-02-05

**Authors:** María V. Jiménez‐Franco, Marc Kéry, Mario León‐Ortega, Francisco Robledano, Miguel A. Esteve, José F. Calvo

**Affiliations:** ^1^ Departamento de Biología Aplicada Universidad Miguel Hernández de Elche Elche Spain; ^2^ Departamento de Ecología e Hidrología Universidad de Murcia Murcia Spain; ^3^ Swiss Ornithological Institute Sempach Switzerland

**Keywords:** Bayesian multispecies occupancy models, cell size, community models, community size, detectability, environmental covariates, forest birds, historical data

## Abstract

New monitoring programs are often designed with some form of temporal replication to deal with imperfect detection by means of occupancy models. However, classical bird census data from earlier times often lack temporal replication, precluding detection‐corrected inferences about occupancy. Historical data have a key role in many ecological studies intended to document range shifts, and so need to be made comparable with present‐day data by accounting for detection probability. We analyze a classical bird census conducted in the region of Murcia (SE Spain) in 1991 and 1992 and propose a solution to estimating detection probability for such historical data when used in a community occupancy model: the spatial replication of subplots nested within larger plots allows estimation of detection probability. In our study, the basic sample units were 1‐km transects, which were considered spatial replicates in two aggregation schemes. We fit two Bayesian multispecies occupancy models, one for each aggregation scheme, and evaluated the linear and quadratic effect of forest cover and temperature, and a linear effect of precipitation on species occupancy probabilities. Using spatial rather than temporal replicates allowed us to obtain individual species occupancy probabilities and species richness accounting for imperfect detection. Species‐specific occupancy and community size decreased with increasing annual mean temperature. Both aggregation schemes yielded estimates of occupancy and detectability that were highly correlated for each species, so in the design of future surveys ecological reasons and cost‐effective sampling designs should be considered to select the most suitable aggregation scheme. In conclusion, the use of spatial replication may often allow historical survey data to be applied formally hierarchical occupancy models and be compared with modern‐day data of the species community to analyze global change process.

## 1 INTRODUCTION

Historical occurrence data represent a common, but underused and valuable source of data that can provide novel insights into how the natural world has changed over human life spans (Tingley & Beissinger, 2009). One of the most relevant uses to which these sources can be put is to forecast species distribution by comparison with past climate conditions (Millar & Woolfenden, 1999). However, due to the complex and variable nature of past occurrence data (museum collections, field notes, etc.), a flexible framework for analysis is needed. Indeed, direct comparison of old, historic, and present‐day data may be complicated or even impossible if the survey methodology has changed or if any other factor that may influence detection probability differently affects old and the new data (Tingley & Beissinger, 2009). Some of the methodological issues involved in using historical data (e.g., limited historic sampling or the inability to control for changes in detectability between sampling periods) can now be explicitly accounted for through occupancy modeling and related quantitative techniques (Kéry & Royle, 2016; Moritz et al., 2008).

Occupancy models provide estimates of occurrence probability for species that are corrected for imperfect detection (Bailey, MacKenzie, & Nichols, 2014; MacKenzie et al., 2006). These models enable us to rigorously evaluate the effects of environmental variables on occupancy probability, mapping species range dynamics (Kéry, Guillera‐Arroita, & Lahoz‐Monfort, 2013; Santika, McAlpine, Lunney, Wilson, & Rhodes, 2014), study the interactions between species (Michel, Jiménez‐Franco, Naef‐Daenzer, & Grüebler, 2016; Yackulic et al., 2014) and evaluate the effects of climate change (Clement, Hines, Nichols, Pardieck, & Ziolkowski, 2016). Multispecies occupancy models are a more complex framework, aimed at estimating total community richness (Dorazio & Royle, 2005; Dorazio, Royle, Söderström, & Glimskär, 2006; Kéry & Royle, 2008) and few studies have evaluated the effects of different habitats (Zipkin, DeWan, & Royle, 2009) and range shift over two different periods (Moritz et al., 2008; Tingley & Beissinger, 2013). The fundamental idea behind the multispecies modeling approach is that collective community data can inform the occurrence probabilities for all observed species, even those that are rare or elusive, and allow for occurrence estimation of species that were never observed in the sample plots (Zipkin et al., 2009). Thus, a multispecies approach can provide more precise estimates of species richness, while accounting for variation in occurrence and detection among species, which is a useful tool to inform which species respond in a comparable manner to habitat changes (Russell et al., 2009). Moreover, in order to separately estimate occupancy and detection probability, it is typically necessary to have replicated observations from at least some of the sites considered in a study (MacKenzie et al., 2002, 2006). There are several ways to collect information about species detectability: temporal replicates, records collected by multiple independent observers, multiple independent detection methods or by spatial subsampling of a site (MacKenzie & Royle, 2005). Most studies so far have used temporal replicates at a site, that is, multiple visits (MacKenzie & Royle, 2005), for example, within one breeding season (León‐Ortega, Jiménez‐Franco, Martínez, & Calvo, 2017). However, spatial replicates at a given site may be a more efficient protocol for large areas (Karanth et al., 2011), or when there are budget constraints (Martínez‐Martí, Jiménez‐Franco, Royle, Palazón, & Calvo, 2016). Spatial replicates are defined as surveyed points or transects that are nested in what is considered a site, such as a grid cell. Such nested subsamples have great potential for mapping species distributions using occupancy modeling over large areas (Srivathsa, Puri, Kumar, Jathanna, & Karanth, 2018). The use of spatial replication in occupancy modeling assumes that the species of interest have a nonzero probability of occurring in each spatial replicate, given that they occur somewhere in the larger grid cell, that is, spatial closure, and uniform availability of the species for detection (Charbonnel et al., 2014; Hines et al., 2010; Kendall & White, 2009). This typically means that individuals may move widely within the latter or that there is suitable habitat for each within the area of every spatial replicate. The topic of spatial subsampling has recently received growing attention, so studies have analyzed and compared spatial versus temporal replicates (Charbonnel et al., 2014), as well as the comparison among different data sources in the same system (Srivathsa et al., 2018).

Documenting range shifts is an integral part of understanding how species and communities have responded to past environmental change, and occupancy models require the same sampling design in the old and the new period if valuable comparisons are to be made. Few studies have used historical data in occupancy models (Eaton, Hughes, Hines, & Nichols, 2014; Moritz et al., 2008; Tingley & Beissinger, 2013), probably due to the lack of temporal replicates in the historical surveys, which precludes the estimation of detectability. When there are no temporal replicates, it is useful to consider spatial subsampling of a site to obtain information about detection probability at a scale larger than the fundamental survey unit. For example, the North American Breeding Bird Survey (Robbins, Bystrack, & Geissler, 1986) consists of roadside surveys, each composed of 50‐point counts spaced at 800‐m intervals. Such surveys could be used to evaluate occupancy by partitioning each site (survey route) into spatial subunits (detection/nondetection of species at single stops or pooled across groups of stops; Kendall & White, 2009). Sadoti, Zuckerberg, Jarzyna, and Porter (2013) aggregated basic atlas survey quadrats into "sites" containing two to four contiguous quadrats, each quadrat serving as a spatial replicate for the analysis of occupancy and detection at site level.

In this study, we use a historical data set from an avian community composed of 1‐km transects surveyed in 1991 and 1992. These transects are grouped as spatial replicates nested within a site so that it is possible to fit occupancy models to correct our inferences on species distributions for imperfect detection. An important aspect for aggregating the original survey units (such us points or transects) is the definition of the site. Although a patch of homogeneous habitat could be considered the most appropriate way of defining a site, grouping spatial replicates in a grid cell is quite useful, since environmental data are typically available in grid cells covering entire geographical areas, for example, from GIS databases (MacKenzie & Royle, 2005), which allows mapping the distribution of every species in the community (Budic, Didenko, & Dormann, 2016). Another important aspect is the selection of the size of a site, which varies in occupancy studies in relation to home range sizes (Efford & Dawson, 2012), and has implication in species distribution models (Yackulic & Ginsberg, 2016). The concept of plot size for the design of occupancy studies in continuous habitat is itself somewhat complex (Efford & Dawson, 2012). The properties of the variable occupancy differ greatly when plots are very large or very small relative to home range size. On the one hand, MacKenzie and Royle (2005) stated that “For a species with relatively large home ranges compared with the size of the sampling units, the proportion of area used over a longer timeframe may be close to 100% even though population size is very small.” On the other hand, plots smaller than a home range would violate the assumption of closure (i.e., constant occupancy) between replicate samples. According to Charbonnel et al. (2014), spatial scale of the sampling sites must be the same as the home range of the species (or as close to it as possible). Therefore, special attention should be paid in community models, where different species may have different sizes of home ranges. Nevertheless, plot size is usually seen as a design variable under the control of the investigator (Efford & Dawson, 2012).

Environmental data from the WorldClim database have small spatial resolution (30‐s latitude/longitude, i.e., 0.93 × 0.93 = 0.86 km^2^ at the equator), so it is possible to resample for different sizes, depending on the study species, the dimensions of the study area, and the specific aspects of the spatial subsampling of a site; for example, length or distribution of transects in a study area (Lipsey, Naugle, Nowak, & Lukacs, 2017). Based on our sample dataset of 1‐km transects, the spatial resolution of 30 s is too small to grouping these spatial replicates, so the double and triple of this spatial resolution of 30 s may be aggregation schemes with the finest potential spatial scales for the size of the site (hereafter, aggregation schemes AS2x2 and AS3x3).

To make an informed choice for the design of a modern‐day bird survey, we fit community occupancy models to our historical data considering 1‐km transects as spatial replicates that are nested within of larger sites defined as grid cells. The aims are as follows: (a) to fit a community occupancy model to a classical bird survey; (b) to compare the relative independency of estimates of species detectability and occupancy between two aggregation schemes corresponding to two different cell site resolutions of potential use in our study area (AS2x2 and AS3x3); (c) to evaluate the influence of environmental variables (forest cover, temperature, and precipitation) on the occurrence of each member in the avian community. We hypothesized that a higher percentage of forest cover may have a positive influence on occupancy probability for the bird community (Gil‐Tena, Saura, & Brotons, 2007; Zipkin, Royle, Dawson, & Bates, 2010). Regarding weather conditions, sites with a higher average temperature and a lower average precipitation could decrease bird occupancy due to climate constrains in Mediterranean semiarid areas (Garrido, Palenzuela, Bañón, & García, 2015; Zuckerberg et al., 2011). This study emphasizes the importance of grouping historical field surveys data within a site, in order to use methods that properly account for imperfect species detection. Moreover, we focus on evaluating the effects of environmental and climatic variables on occupancy of bird species in a Mediterranean region of varying climate sensitivity (Garrido et al., 2015).

## 2 MATERIALS AND METHODS

### 2.1 Study area and species

This study was developed in the region of Murcia (SE Spain) with an area of 11,317 km^2^ and a semiarid Mediterranean climate, where during the last five decades (1961–2014), the annual temperature has been 16.7°C, with a tendency to increase by 0.135°C per decade (Garrido et al., 2015). The annual precipitation for this period was 310 mm/year on average, with a wide degree of variation. A clear tendency related to climate change is the decrease in the number of days with snow in the coldest part of the study area (NW), falling from 20 (at the end of the 1960s) to 10 days (in the 2010s; Garrido et al., 2015). The wide climatic gradient in the study area means that it harbors several types of ecosystem that conform to an ecotone between the Mediterranean and arid subtropical: semi‐desert areas, Mediterranean scrub, and coniferous forest (Esteve et al., 2015). In compliance with the European Birds and Habitats Directives, 22 Special Protection Areas for birds have been designated in the region (Abellán, Martínez, Palazón, Esteve, & Calvo, 2011). As a result of the climatic gradient and the different ecosystems, the bird community is diverse, with a total of 339 bird species cataloged from 24 orders and 69 families (Calvo et al., 2017).

**Figure 1:**
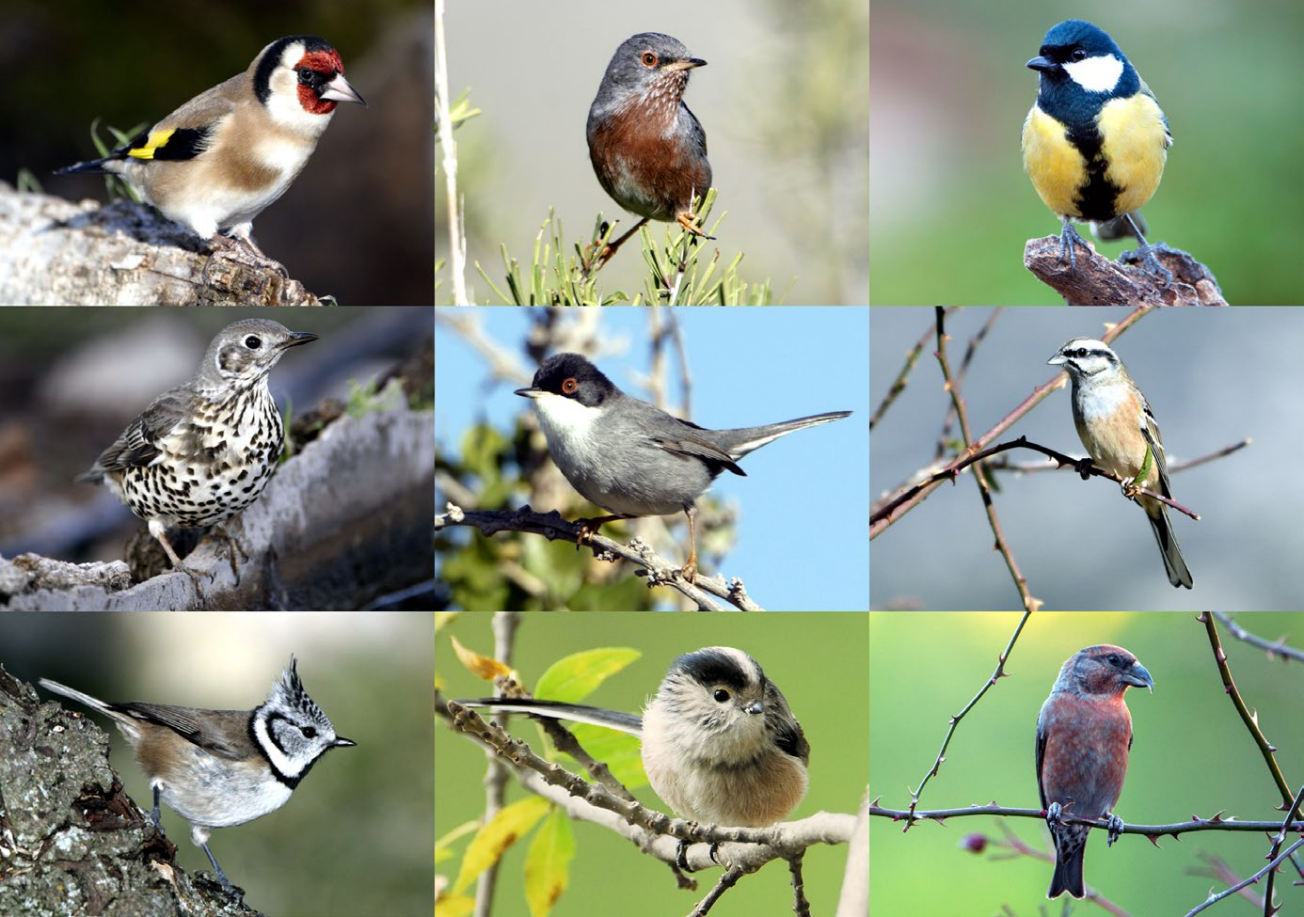
Nine representative bird species of the study area. From left to right, and top to bottom: *Carduelis carduelis*, *Sylvia undata*, *Parus major*, *Turdus viscivorus*, *Sylvia melanocephala*, *Emberiza cia*, *Lophophanes cristatus*, *Aegithalos caudatus,* and *Loxia curvirostra*. Photograph credit: Carlos González Revelles

This study focuses on bird species inhabiting Mediterranean forest ecosystems in the region of Murcia (Figure 1). These forest areas are dominated by one tree species, the Aleppo pine (*Pinus halepensis*), a conifer that may reach up to 22 m in Mediterranean areas (Mitsopoulos & Dimitrakopoulos, 2014), having opened areas comprised of Mediterranean scrubs. A total of 73 avian species were recorded (Supporting Information Table S1), being most of them passerines, and the most representative families Sylviidae, Turdidae, Fringillidae, and Paridae. These four families encompass more than a third of the forest bird community (31 species), being indicative of different status of forest maturity and including representatives of relevant trophic and functional guilds (granivores, frugivores, seed dispersers, etc.), as well as of different zoogeographical origins, for example, boreal versus Mediterranean species (Blondel, Aronson, Bodiou, & Boeuf, 2010).

### 2.2 Study sampling: forestry plan

**Figure 2:**
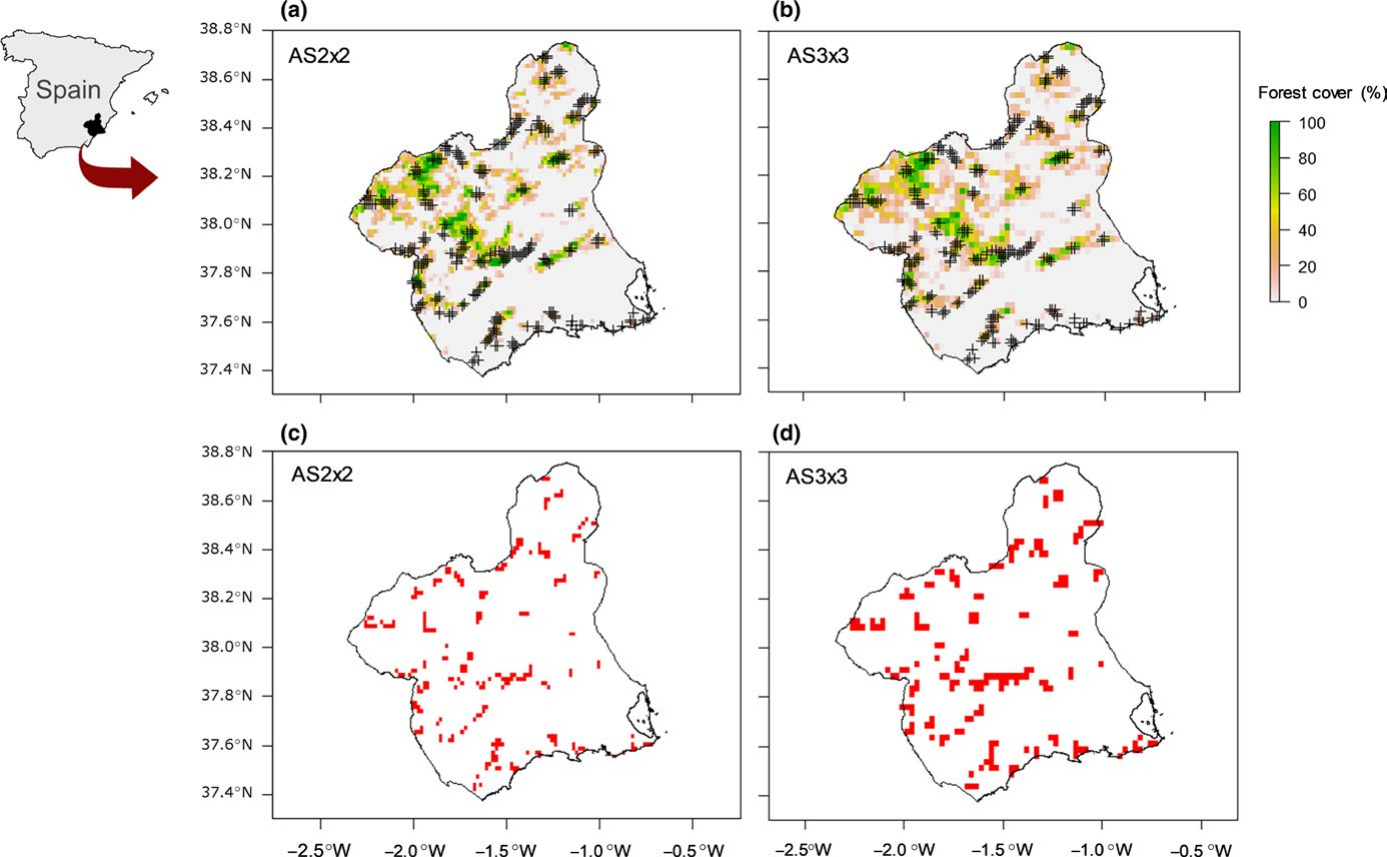
Distribution of 1‐km bird transects conducted in Mediterranean forest areas in the study area (region of Murcia, SE Spain), considering two different grid sizes: (a) aggregation scheme AS2x2 (60‐s latitude/longitude) and (b) aggregation scheme AS3x3 (90‐s latitude/longitude). Cross symbols represent the mean positions for the 377 transects, which were grouped as spatial replicates considering two different aggregation schemes for sites: (c) 246 sites for AS2x2; (d) 185 sites for AS3x3

Between 1991 and 1992, an intensive monitoring program was conducted as part of a forestry plan in all the region of Murcia with the aim of characterizing breeding bird communities as a basis for assessing the state of the region forest heritage (Esteve, 1991). The classical surveys consist of 377 1‐km transects covering the whole study area and distributed randomly in forested areas (Figure 2a,b) during the reproductive period (May to July; Hernández & Barberá, 1997). Each transect was conducted by walking and recording the number of each species detected (by sight or song), giving a total of 73 forest bird species recorded (Supporting Information Table S1).

### 2.3 Data resampling and environmental covariates

We nested the 377 1‐km transects, considering them as spatial observations replicated within the site, which are necessary for estimating detection probability. Sites are defined as grid cells that together cover the whole study area. This composition of sites along with environmental covariates is useful for estimating site‐level occupancy (Sadoti et al., 2013). Since the resolution of the cells (sampling unit of the site) might vary depending on the spatial scale and species being studied, two potential aggregation schemes were chosen, with different grid cell sizes, an aggregation scheme with a grid size of 60 × 60 and 90 × 90 s, (AS2x2 and AS3x3, respectively). Based on the species of bird community (most of them passerines with small home ranges; Rechetelo, Grice, Reside, Hardesty, & Moloney, 2016), both sizes of grid cell may be suitable for evaluating the effects of environmental covariates on bird species occupancy at regional scale (Kéry et al., 2013; Lipsey et al., 2017). The aggregation scheme AS2x2 grouped from 1 to 5 transects per site, giving a total of 246 cell sites with observations (Figure 2a,c). The aggregation scheme AS3x3 grouped from 1 to 7 transects per site, giving a total of 185 cell sites with data in the region of Murcia (Figure 2b,d).

The percentage of forest cover (FOREST) was estimated from the CORINE Land Cover 1990 map (http://centrodedescargas.cnig.es), using data from 0% to 100% (mean = 28.51%). Climate variables were the annual mean temperature (TEMP) and the annual precipitation (PREC) obtained from the average monthly climate data of temperature and precipitation for the period 1960–1990, which were downloaded from the global dataset WorldClim version 1.4 (Hijmans, Cameron, Parra, Jones, & Jarvis, 2005; http://www.worldclim.org/version1). The annual mean temperature ranged from 10.9°C to 18.3°C and from 10.72°C to 18.13°C for AS2x2 and AS3x3, respectively. The annual precipitation ranged from 275 to 580 mm and from 274.1 to 596.0 mm for AS2x2 and AS3x3, respectively. GIS analyses were carried out with the *raster* package (Hijmans, 2016) in R 3.3.2 (R Core Team, 2016).

### 2.4 Hierarchical models for communities

Two Bayesian multispecies occupancy models (Dorazio & Royle, 2005; Dorazio et al., 2006), one for each cell site aggregation scheme (AS2x2 and AS3x3) were fitted. These models are an extension of the single species site occupancy model (MacKenzie et al., 2002), whereby the hierarchical structure combines community and species‐level attributes within a single analytical framework. The hierarchical models are composed of the ecological process (governed by occupancy probability) and the observational process (governed by detectability probability). Data are compiled as a 2 × 2 matrix Ysum with *i* rows by *k* columns, corresponding to sites and species, respectively. The number of spatial replicates *j* for each site *i* where the species *k* was observed is quantified in the matrix Ysum. The ecological process assumes that site‐specific occupancy (i.e., “true” presence/absence) for species *k* = 1, 2,…, *N* at site *i = *1, 2,…, *Nsite*, denoted *z*(*i,k*), where *z*(*i,k*) = 1 if species *k* occurs in site *i* and is zero otherwise. The model for occurrence is specified as *z*(*i,k*) ~ Bern(*ψ_i,k_*) where (*ψ_i,k_*) is the probability that species *k* occurs at site *i*. The true occurrence is imperfectly observed, and we define the detection model for species *k* at site *i* in replicate *j* as Ysum(*i,k*) ~ Binomial(*p_ik_*·*z*(*i,k*)), where *p_i,k_* is the detection probability of species *k *for the *j*th spatial replicate at site *i*, given that species *k* is in fact present at site *i* (Zipkin et al., 2009). In the simplest specification of the model, the occurrence and detection probabilities are composed of species‐specific effects and site‐level effects (Dorazio et al., 2006; Kéry & Royle, 2016). Extensions of this basic model have explicitly incorporated landscape characteristics into the probability of occupancy (Kéry & Royle, 2009, 2016; Kéry, Royle, & Schmid, 2008; Zipkin et al., 2009). We followed this approach, and modeled the occurrence probability for species *k* at site *i* by incorporating site‐specific habitat characteristics and environmental covariates. Linear and quadratic effects of percentage forest covariate and temperature were included. The model also included a linear effect of precipitation for each site. All habitat variables were standardized. Therefore, we defined the probability of occupancy as follows:

$$\text{logit}\left(\psi_{i,k}\right)∼\text{Normal}\left(\mu_{lpsi,i,k},\sigma_{lpsi,i,k}^{2}\right),$$

$$\begin{array}{c} \mu_{lpsi,i,k}=\text{delta}0_{k}+\text{delta}1_{k}\cdot {\text{FOREST}}_{i}+\text{delta}2_{k}\cdot {\text{FOREST}}_{i}^{2}+\text{delta}3_{k}\cdot {\text{TEMP}}_{i}\\ +\text{delta}4_{k}\cdot {\text{TEMP}}_{i}^{2}+\text{delta}5_{k}\cdot {\text{PREC}}_{i}. \end{array}$$

The inverse‐logit of delta0*_k_* is the occurrence probability for species *k* at a site with “average” habitat characteristics. The coefficients from delta1*_k_* to delta5*_k_* are the effects of the percentage of forest cover (linear and squared), the annual mean temperature (linear and squared), and the annual precipitation, for species *k,* respectively. We assumed that detection probabilities varied depending on the species but were not influenced by survey characteristics: $\text{logit}\left(p_{k}\right)∼\text{Normal}\left(\mu_{\left(lp,k\right)},\sigma_{\left(lp,k\right)}^{2}\right)$. Moreover, we have not modeled the influence of temporal effects (e.g., Julian date) on detectability since it has a more relevant interest in temporal replicates rather than spatial replicates (Kéry et al., 2013; Zipkin et al., 2010). As observations were sparse for many species in the sample, estimating all of these parameters would not have been possible if the data were analyzed on a species‐by‐species basis. Therefore, we added an additional hierarchical component of the model by assuming that the species‐level parameters were random effects, each governed by community‐level “hyper‐parameters.” For example, a community response (mean across species) for a site and standard deviation (among species) were estimated, so that the hyper‐parameters are simply the mean and variance for each covariate, as measured across species (Kéry & Royle, 2016). The two models for each aggregation scheme (AS2x2 and AS3x3) were fitted using JAGS (Plummer, 2003), run in R 3.3.2 (R Core Team, 2016) with the package *jagsUI* (Kellner, 2015), using uninformative priors, three chains, 15,000 iterations, and a burn‐in of 5,000 iterations and a thin rate of 2 (see R and JAGS code in Supporting Information Appendix S1). Convergence was assessed by examining the Rhat values for each parameter estimate (Brooks & Gelman, 1998). We present posterior means and standard deviations for point estimates and the Bayesian analog to a standard error.

### 2.5 Model comparisons

In each aggregation scheme (AS2x2 and AS3x3), we evaluated the estimates of detectability and occupancy for each species, as well as the estimates of regression coefficients for predictor variables. A linear correlation was performed among species estimates of detectability and occupancy, comparing both aggregation schemes.

## 3 RESULTS

A total of 377 spatial transects were grouped for each aggregation scheme (AS2x2 and AS3x3) into 246 and 185 sites, respectively (Table 1, Figure 2). The distribution of spatial transects varied between both aggregation schemes based on the dimensions of cell sites (Supporting Information Figure S1). A total of 73 species were observed for both aggregation schemes, with a mean of observed species richness per cell site of approximately 15 and 17 species for AS2x2 and AS3x3, respectively (Supporting Information Figure S2). The estimated community size for each sampling site was 27.73 ± 3.88 and 29.35 ± 4.13 species for AS2x2 and AS3x3, respectively (*Nsite *= 246, Supporting Information Table S2; *Nsite *= 185, Supporting Information Table S3). The estimated richness for each site is shown in Supporting Information Tables S2 and S3. The estimates of occupancy for the whole community (*lpsi* mean) are 0.39 and 0.41 for AS2x2 and AS3x3, respectively, and the mean of detection probability (*lp* mean) was 0.40 and 0.35 for AS2x2 and AS3x3, respectively (Supporting Information Tables S2 and S3).

**Table 1:** Distribution of 1‐km bird transects in the study area (region of Murcia, SE Spain) grouped as spatial replicates considering two different grid sizes of 60 and 90 s latitude/longitude (aggregation schemes AS2x2 and AS3x3, respectively)

Aggregation schemes	*N*. transects per site							Total of sites	Total of transects
	1	2	3	4	5	6	7		
AS2x2	148	71	22	4	1	0	0	246	377
AS3x3	83	50	24	22	3	2	1	185	377

**Figure 3:**
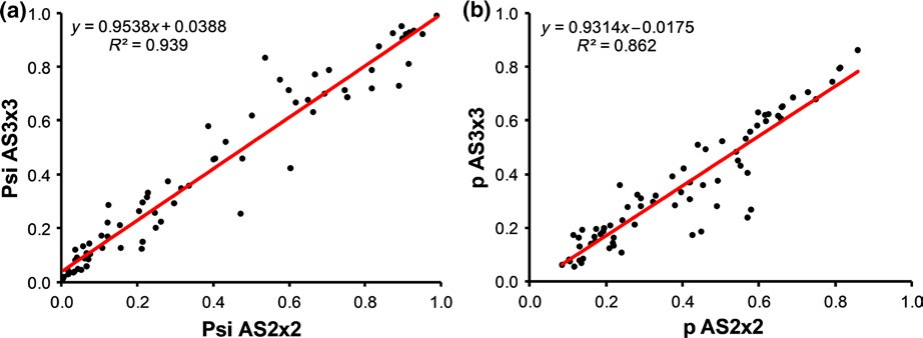
Mean of estimates of (a) occupancy probability *Psi* and (b) detection probability *p* for bird species under the two aggregation schemes AS2x2 and AS3x3 (*x* and *y* axes, respectively)

The result of community occupancy models for each species‐specific shows the estimates of detectability and occupancy; as can be seen they vary greatly among species for both aggregation schemes (Supporting Information Figure S3). Whereas *Serinus serinus* is the species with the highest mean *p* and *psi* in both aggregation schemes, the species with the lowest probability of occupancy was *Alauda arvensis* and with the lowest probability of detection *Motacilla alba* for AS2x2 and *Phylloscopus collybita* for AS3x3 (Supporting Information Table S4). The estimates of detection and occupancy probability for each avian species comparing the aggregation schemes are shown in Figure 3, where both estimates are closely correlated between the two aggregation schemes (*r* = 0.929 for *p*; *r* = 0.969 for *Psi*).

**Figure 4:**
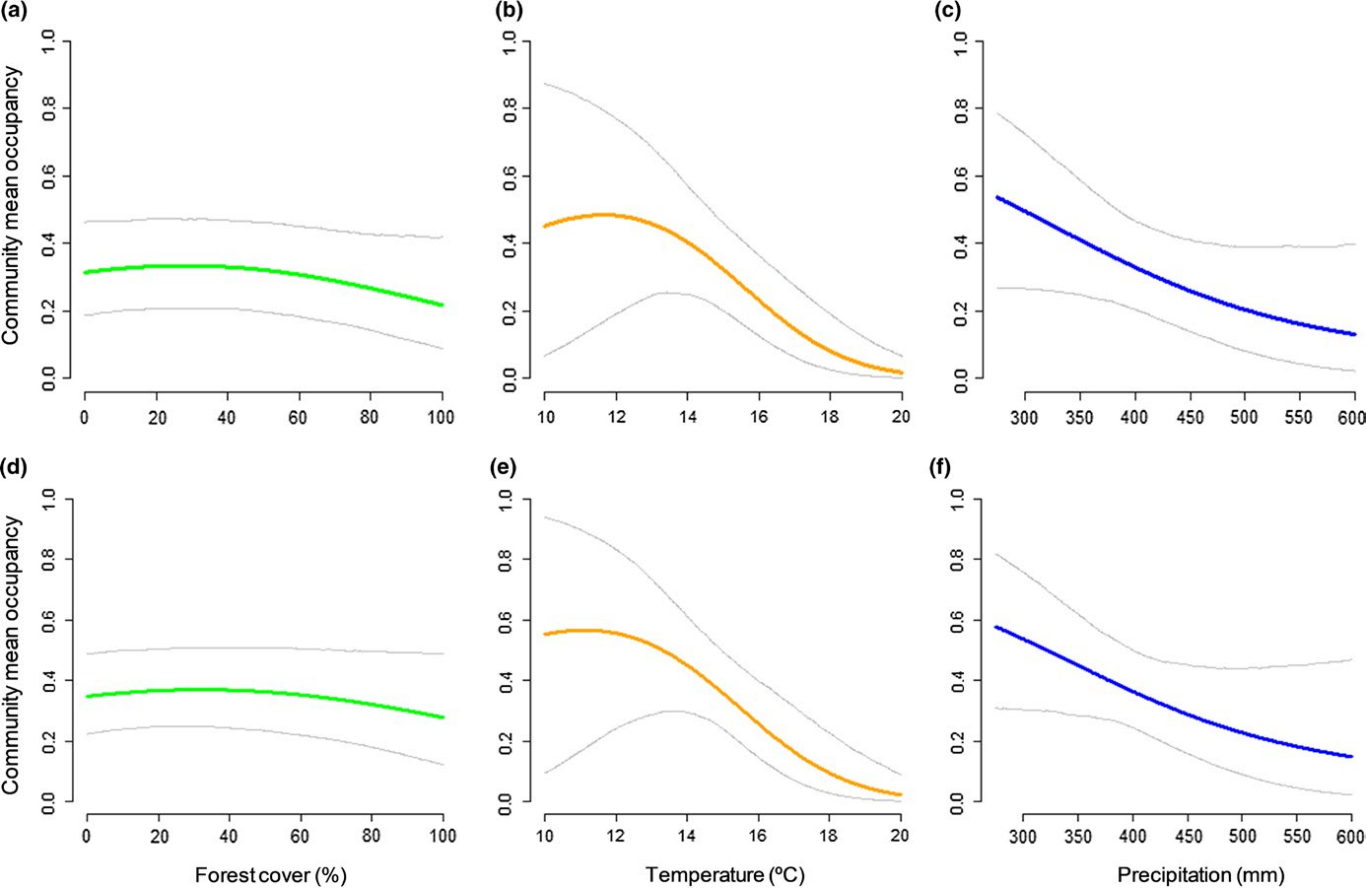
Community response of bird species occupancy probability to forest cover, temperature and precipitation for the aggregation schemes AS2x2 (a–c) and AS3x3 (d–f). Gray lines show 95% CI of the community mean

**Figure 5:**
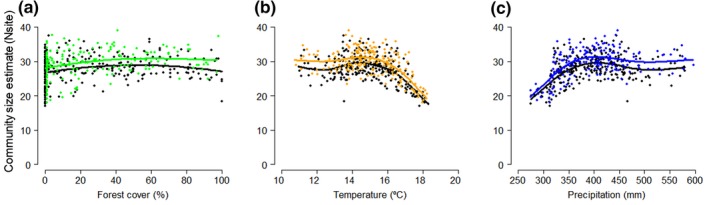
Relationships between the number of bird species (community size, *Nsite*) and the covariates of each sampled site: (a) forest cover, (b) temperature, and (c) precipitation. Each point represents the richness of each cell site surveyed (*n = *246 and *n = *185 for the aggregation schemes AS2x2 and AS3x3, respectively). Lines represent splines smooth. Comparison of aggregation schemes shown in different colors: AS2x2 (black) and AS3x3 (green, yellow, and blue for the percentage of forest cover, annual mean temperature, and annual precipitation, respectively). Note slight offset of color points in the *x* direction for the aggregation scheme AS3x3

To evaluate environmental effects on the bird community, the estimates of community occupancy in relation to the percentage of forest cover, the annual mean temperature, and annual precipitation are shown in Figure 4 for each aggregation scheme: AS2x2 (Figures 4a‐c) and AS3x3 (Figures 4d‐f). As can be seen, the effects of the forest cover and environmental covariates have similar tendencies between schemes; Supporting Information Figure S4 depicts the tendencies for each species. The mean of species richness for each cell site is shown along the gradient of the environmental covariates for all sites (Figure 5). The estimate is slightly higher for the aggregation scheme with the greater cell site size (AS3x3), especially in cell sites with extreme environmental gradients—high percentage of forest cover and annual precipitation, and low annual mean temperature (Figure 5a–c).

## 4 DISCUSSION

This study offers a new look at the use of historical data by means of considering them as spatial replicates that are nested within a site, and develops community occupancy models. This approach can be extrapolated to other species and areas, where valuable data from historical bird surveys can be used (Tingley & Beissinger, 2009). Classical bird surveys were designed previously to the development of hierarchical occupancy models (MacKenzie et al., 2006), so we grouped these transects into larger cells to serve as spatial replicates for a site defined in this way. This framework allows community occupancy models to be fitted, providing individual estimates for occupancy and species richness and accounting for imperfect detection in classical monitoring programs. Moreover, the effects of habitat covariates on the estimated parameters must be considered to study the environmental response of bird communities (Tingley & Beissinger, 2009; Zipkin et al., 2009). To the best of our knowledge, this is the first study that specifically was able to make use of historical data by means of spatial replicates to make inference about a large community of birds and their habitat associations. Future studies will hopefully “rediscover” historical occurrence data and elucidate on how communities, populations, and species have shifted over temporal scales (Tingley & Beissinger, 2009). It is also important to consider that the ecological system must be combined with a good understanding of the statistical principles behind sampling in order to improve the value of monitoring programs (Karanth et al., 2011). Furthermore, the effectiveness of related management actions is especially relevant in the context of ecology and conservation, fields where resources are often fairly limited, and can be increased by applying spatial replicates in sample fields involved in monitoring species at large landscape scales (Martínez‐Martí et al., 2016; Srivathsa et al., 2018). It is also important to highlight that in order to compare the range shift from historical data and compare occupancy and detectability among years, we should consider the field design established, so future surveys will be conducted following similar protocol of the first (Yackulic & Ginsberg, 2016). Moreover, in order to establish probabilities of extinction and colonization without bias, it would be necessary to use the same size of aggregation scheme by including surveys in the same cell sites for the following period (Peach, Cohen, & Frair, 2017; Sadoti et al., 2013). Moreover, the mixture of spatial and temporal replicates to estimate detection may also induce bias, so this bias can be removed by choosing sampling locations with replacement, or if the species is highly mobile over a short period of time (Kendall & White, 2009).

During the development of this new design, the question emerged concerning the potential size of grid for aggregating spatial transects in a cell site. As we stated in the introduction section, the finest cell site resolution may be of 30 × 30 s (i.e., 0.86 km^2^), so we generate two potential aggregation schemes of 60 × 60 and 90 × 90 s (AS2x2 and AS3x3). The results for models using the aggregation schemes AS2x2 and AS3x3 showed that estimates of occupancy and detectability were highly correlated between both aggregation schemes. Therefore, we consider that both aggregation schemes are equally useful to develop this hierarchical framework in the future bird surveys. In other words, and following our previous hypothesis, our results show relative independence between the two sizes of cell sites proposed. Therefore, ecological reasons and cost‐effective sampling designs should be considered to select the more suitable aggregation scheme in the future studies. Nevertheless, models with data augmentation could be more precise for estimating community size (Zipkin et al., 2009), although this was not the aim of this study. Our study design will allow us to implement a dynamic framework, by comparing past bird surveys with modern surveys designed for the same conditions and also estimating parameters that govern change in species presence/absence, for example, probabilities of extinction and colonization (Dorazio, Kéry, Royle, & Plattner, 2010; MacKenzie, Nichols, Hines, Knutson, & Franklin, 2003). This application is relevant for Breeding Bird Atlas Projects (Peach et al., 2017). Another recent extension developed for community occupancy models is the extension of hierarchical models to multi‐scale habitat selection (Lipsey et al., 2017) and across multiple regions of interest (e.g., reserves or biomes; Sutherland, Brambilla, Pedrini, & Tenan, 2016), allowing the estimation of region‐specific community size.

Another relevant property of community occupancy models is that they allow occupancy probability to be obtained in relation to environmental covariates and to analyze climate change effects (Clement et al., 2016). Our results show that a higher percentage of forest cover reduces slightly the mean occupancy of bird communities. Moreover, when environmental covariates (annual mean temperature and annual precipitation) increase, the mean occupancy of bird species is reduced drastically for both aggregation schemes. This result does not agree with a previous study of Tayleur et al. (2015), where Swedish birds are tracking by temperature but not by rainfall. It is probably due to the different climate conditions between both avian communities. These aspects need to be borne in mind in light of the changing meteorological conditions in SE Spain at nowadays, since it is an ideal area to study climate change effects and bird distribution in semiarid ecosystems (Esteve et al., 2015). Therefore, this study could be considered as a pilot study before evaluating climate change effects in southeastern Spain through the development of modern bird surveys. This application of community occupancy models to evaluate climate change effects has been explored recently (Tingley & Beissinger, 2013), although spatial replicates have only been used in one mammal study (Moritz et al., 2008). Studies about community‐level responses to environmental variations have been developed along with new statistical tools (Kéry & Royle, 2016) which allow the estimate of nondetected species through data augmentation (Dorazio et al., 2006), estimates of abundance (Yamaura et al., 2016), the development of community dynamic models (Dorazio et al., 2010), and even the combination of trait data with phylogenetic data (taxonomy identity; Ovaskainen et al., 2017). In this line, future research should focus on developing these mechanistic tools for the study of community species, optimizing sampling effort, and allowing managers to obtain valuable ecological information on wildlife species.

## CONFLICT OF INTEREST

None declared.

## AUTHOR CONTRIBUTION

MVJF, JFC, MK, and MAE conceived the ideas and designed methodology; MAE, FR, and MLO collected the data; MVJF, MK, and JFC analyzed the data; MVJF led the writing of the manuscript. All authors contributed critically to the drafts and gave final approval for publication.

## Data Availability

Data available from the Dryad Digital Repository: https://doi.org/10.5061/dryad.8sf5v66.
