# Supplementary material for: Use of classical bird census transects as spatial replicates for hierarchical modeling of an avian community
Source: Ecol Evol. 2019 Feb 5;9(2):825–35. doi: 10.1002/ece3.4829 (PMC6362445; doi:10.1002/ece3.4829)
Supplement: Supplementary file 1 [file ECE3-9-825-s001.docx]

**Use of classical bird census transects as spatial replicates for hierarchical modelling of an avian community**

María V. Jiménez-Franco, Marc Kéry, Mario León-Ortega, Francisco Robledano, Miguel A. Esteve and José F. Calvo

**Appendix S1. R code for hierarchical models for communities.**

## R and WinBUGS/JAGS Code for species occupancy model reported in:

## "Use of classical bird census transects as spatial replicates for hierarchical modelling of an avian community"

## by María V. Jiménez-Franco, Marc Kéry, Mario León-Ortega, Francisco Robledano, Miguel A. Esteve and José F. Calvo

## Last edited by María V. Jiménez-Franco on 1st June 2017

#Load the correct library

library(jagsUI)

# Read in the data

#set number of sites, year, and repetitions

str(bdata <- list(Ysum = Ysum, nsite = nrow(Ysum), nrep = J, nspec = ncol(Ysum), forest = forest, temp = temp, prec = prec) )

##################### Psi(forest2+Temp2+Prec), p(.)

### Next, the model with 3 site-level covariates

# ----------------------------------------------

# For illustration, fit linear and quadratic effects of forest cover, temperature and precipitation

# Bundle and summarize data

str(bdata <- list(Ysum = Ysum, nsite = nrow(Ysum), nrep = J, nspec = ncol(Ysum), forest = forest, temp = temp, prec = prec) )

# Specify model in BUGS language

sink("model_AS.txt")

cat("

model {

# Priors

# Priors for species-specific effects in occupancy and detection

for(k in 1:nspec){

lpsi[k] ~ dnorm(mu.lpsi, tau.lpsi) # Hyperparams describe community

lp[k] ~ dnorm(mu.lp, tau.lp)

beta1[k] ~ dnorm(mu.beta1, tau.beta1) # coefficients for effect of forest (linear)

beta2[k] ~ dnorm(mu.beta2, tau.beta2) # coefficients for effect of forest (squared)

beta3[k] ~ dnorm(mu.beta3, tau.beta3) # coefficients for effect of temp (linear)

beta4[k] ~ dnorm(mu.beta4, tau.beta4) # coefficients for effect of temp (squared)

beta5[k] ~ dnorm(mu.beta5, tau.beta5) # coefficients for effect of prec (linear)

}

# Hyperpriors

# For the model of occupancy

mu.lpsi <- logit(mean.psi) # Occupancy intercepts

mean.psi ~ dunif(0, 1)

tau.lpsi <- pow(sd.lpsi, -2)

sd.lpsi ~ dunif(0, 5) # as always, bounds of uniform must be chosen by trial and error

mu.beta1 ~ dnorm(0, 0.01) # Linear effect of forest

tau.beta1 <- pow(sd.beta1, -2)

sd.beta1 ~ dunif(0, 5)

mu.beta2 ~ dnorm(0, 0.01) # Quadratic effect of forest

tau.beta2 <- pow(sd.beta2, -2)

sd.beta2 ~ dunif(0, 5)

mu.beta3 ~ dnorm(0, 0.01) # linear effect of temp

tau.beta3 <- pow(sd.beta3, -2)

sd.beta3 ~ dunif(0, 5)

mu.beta4 ~ dnorm(0, 0.01) # quadratic effect of temp

tau.beta4 <- pow(sd.beta4, -2)

sd.beta4 ~ dunif(0, 5)

mu.beta5 ~ dnorm(0, 0.01) # linear effect of prec

tau.beta5 <- pow(sd.beta5, -2)

sd.beta5 ~ dunif(0, 5)

# For the model of detection

mu.lp <- logit(mean.p)

mean.p ~ dunif(0, 1)

tau.lp <- pow(sd.lp, -2)

sd.lp ~ dunif(0, 5)

# Ecological model for true occurrence (process model)

for(k in 1:nspec){

for (i in 1:nsite) {

logit(psi[i,k]) <- lpsi[k] + beta1[k] * forest[i] + beta2[k] * pow(forest[i], 2) +

beta3[k] * temp[i] + beta4[k] * pow(temp[i],2) +

beta5[k] * prec[i]

z[i,k] ~ dbern(psi[i,k])

}

}

# Observation model for replicated detection/nondetection observations

for(k in 1:nspec){

for (i in 1:nsite){

logit(p[i,k]) <- lp[k]

mu.p[i,k] <- z[i,k] * p[i,k]

Ysum[i,k] ~ dbinom(mu.p[i,k], nrep[i])

}

}

# Derived quantities

for (i in 1:nsite){

Nsite[i] <- sum(z[i,]) # Number of occurring species at each site

}

}

",fill = TRUE)

sink()

# Initial values

zst <- Ysum

zst[zst > 1] <- 1

inits <- function() list(z = zst, mean.psi = runif(1), mean.p = runif(1))

# List of params estimated

params <- c("mean.psi", "mu.lpsi", "sd.lpsi",

"mu.beta1", "sd.beta1", "mu.beta2", "sd.beta2", "mu.beta3", "sd.beta3",

"mu.beta4", "sd.beta4", "mu.beta5", "sd.beta5",

"mean.p", "mu.lp","sd.lp", # These are the hyperparams

"lpsi", "beta1", "beta2", "beta3", "beta4", "beta5", "lp", # Reg coefficients for the 73 species

"Nsite") # Derived quant

# MCMC settings

na <- 1000 ; ni <- 15000 ; nt <- 10 ; nb <- 5000 ; nc <- 3

# Run JAGS, check convergence and summarize posteriors

fmAS <- jags(bdata, inits, params, "model_AS.txt", n.adapt = na, n.chains = nc, n.thin = nt, n.iter = ni, n.burnin = nb, parallel = TRUE)

par(mfrow = c(2, 2), mar = c(5,4,3,2)) ; traceplot(fmAS)

print(fmAS, 3)
