# Supplementary material for: Use of classical bird census transects as spatial replicates for hierarchical modeling of an avian community
Source: Ecol Evol. 2019 Feb 5;9(2):825–35. doi: 10.1002/ece3.4829 (PMC6362445; doi:10.1002/ece3.4829)

**Use of classical bird census transects as spatial replicates for hierarchical modelling of an avian community**

María V. Jiménez-Franco, Marc Kéry, Mario León-Ortega, Francisco Robledano, Miguel A. Esteve and José F. Calvo

**Supplementary material**

**Table S1.**  List of bird species observed in the 1 km transects conducted in the region of Murcia (SE Spain) between 1991 and 1992.

| **Number of species** | **Scientific name^a^** | **Common name** | **Representative avian family** |
| --- | --- | --- | --- |
| 1 | *Aegithalos caudatus* | Long-tailed Tit | - |
| 2 | *Alauda arvensis* | Eurasian Skylark | - |
| 3 | *Alectoris rufa* | Red-legged Partridge | - |
| 4 | *Anthus campestris* | Tawny Pipit | - |
| 5 | *Athene noctua* | Little Owl | - |
| 6 | *Calandrella brachydactyla* | Greater Short-toed Lark | - |
| 7 | *Calandrella rufescens* | Lesser Short-toed Lark | - |
| 8 | *Carduelis cannabina* | Common Linnet | Fringillidae |
| 9 | *Carduelis carduelis* | European Goldfinch | Fringillidae |
| 10 | *Certhia brachydactyla* | Short-toed Treecreeper | - |
| 11 | *Cettia cetti* | Cetti's Warbler |  |
| 12 | *Chloris chloris* | European Greenfinch | Fringillidae |
| 13 | *Clamator glandarius* | Great Spotted Cuckoo | - |
| 14 | *Columba livia* | Rock Dove | - |
| 15 | *Columba oenas* | Stock Dove | - |
| 16 | *Columba palumbus* | Common Wood Pigeon | - |
| 17 | *Coracias garrulus* | European Roller | - |
| 18 | *Corvus corax* | Northern Raven | - |
| 19 | *Corvus corone* | Carrion Crow | - |
| 20 | *Corvus monedula* | Western Jackdaw | - |
| 21 | *Coturnix coturnix* | Common Quail | - |
| 22 | *Cuculus canorus* | Common Cuckoo | - |
| 23 | *Cyanistes caeruleus* | Blue Tit | Paridae |
| 24 | *Dendrocopos major* | Great Spotted Woodpecker | - |
| 25 | *Emberiza calandra* | Corn Bunting | - |
| 26 | *Emberiza cia* | Rock Bunting | - |
| 27 | *Emberiza cirlus* | Cirl Bunting | - |
| 28 | *Erithacus rubecula* | European Robin | Turdidae |
| 29 | *Erythropygia galactotes* | Rufous-tailed Scrub Robin | Turdidae |
| 30 | *Ficedula hypoleuca* | European Pied Flycatcher | - |
| 31 | *Fringilla coelebs* | Common Chaffinch | Fringillidae |
| 32 | *Galerida cristata/theklae* | Crested/Thekla Lark | - |
| 33 | *Garrulus glandarius* | Eurasian Jay | - |
| 34 | *Hippolais polyglotta* | Melodious Warbler | Sylviidae |
| 35 | *Lanius meridionalis* | Southern Grey Shrike | - |
| 36 | *Lanius senator* | Woodchat Shrike | - |
| 37 | *Lophophanes cristatus* | European Crested Tit | Paridae |
| 38 | *Loxia curvirostra* | Red Crossbill | Fringillidae |
| 39 | *Lullula arborea* | Woodlark | - |
| 40 | *Luscinia megarhynchos* | Common Nightingale | Turdidae |
| 41 | *Melanocorypha calandra* | Calandra Lark | - |
| 42 | *Monticola solitarius* | Blue Rock Thrush | Turdidae |
| 43 | *Motacilla alba* | White Wagtail | - |
| 44 | *Motacilla cinerea* | Grey Wagtail | - |
| 45 | *Muscicapa striata* | Spotted Flycatcher | - |
| 46 | *Oenanthe hispanica* | Black-eared Wheatear | Turdidae |
| 47 | *Oenanthe leucura* | Black Wheatear | Turdidae |
| 48 | *Oenanthe oenanthe* | Northern Wheatear | Turdidae |
| 49 | *Oriolus oriolus* | Eurasian Golden Oriole | - |
| 50 | *Parus major* | Great Tit | Paridae |
| 51 | *Passer domesticus* | House Sparrow | - |
| 52 | *Periparus ater* | Coal Tit | Paridae |
| 53 | *Petronia petronia* | Rock Sparrow | - |
| 54 | *Phoenicurus ochruros* | Black Redstart | Turdidae |
| 55 | *Phylloscopus bonelli* | Western Bonelli's Warbler | Sylviidae |
| 56 | *Phylloscopus collybita* | Common Chiffchaff | Sylviidae |
| 57 | *Pica pica* | Common Magpie | - |
| 58 | *Picus sharpei* | Iberian Green Woodpecker | - |
| 59 | *Pyrrhocorax pyrrhocorax* | Red-billed Chough | - |
| 60 | *Saxicola torquatus* | Common Stonechat | Turdidae |
| 61 | *Serinus serinus* | European Serin | Fringillidae |
| 62 | *Streptopelia turtur* | European Turtle Dove | - |
| 63 | *Sturnus unicolor* | Spotless Starling | - |
| 64 | *Sylvia borin* | Garden Warbler | Sylviidae |
| 65 | *Sylvia cantillans* | Subalpine Warbler | Sylviidae |
| 66 | *Sylvia conspicillata* | Spectacled Warbler | Sylviidae |
| 67 | *Sylvia hortensis* | Western Orphean Warbler | Sylviidae |
| 68 | *Sylvia melanocephala* | Sardinian Warbler | Sylviidae |
| 69 | *Sylvia undata* | Dartford Warbler | Sylviidae |
| 70 | *Troglodytes troglodytes* | Winter Wren | - |
| 71 | *Turdus merula* | Common Blackbird | Turdidae |
| 72 | *Turdus viscivorus* | Mistle Thrush | Turdidae |
| 73 | *Upupa epops* | Hoopoe | - |

^a^ species nomenclature is based on Calvo et al. (2017). Catálogo de las aves de la Región de Murcia (España). DOI: <http://dx.doi.org/10.6018/analesbio.39.02>

**Table S2.** Output of community occupancy model for aggregating the basic surveys (1 km transects) by the aggregation scheme AS2x2. Results include the estimates for community-level parameters, including the covariates for occupancy (beta1: linear effect of forest cover. beta2: quadratic effect of forest cover. beta3: linear effects of temperature. beta4: quadratic effect of temperature. beta5: linear effect of precipitation). Species-level parameters include occupancy (lpsi) and detectability (lp), whose identity for the 73 species is numbered according to Table S1. Site level results include the estimate of community size for the 246 samples sites (Nsite).

|  | **mean** | **sd** | **2.50%** | **97.50%** |
| --- | --- | --- | --- | --- |
| **Community-level parameters** | | |  |  |
| mean.psi | 0.335 | 0.068 | 0.207 | 0.472 |
| mu.lpsi | -0.703 | 0.314 | -1.342 | -0.113 |
| sd.lpsi | 2.295 | 0.243 | 1.864 | 2.801 |
| mu.beta1 | -0.001 | 0.104 | -0.208 | 0.201 |
| sd.beta1 | 0.638 | 0.103 | 0.458 | 0.868 |
| mu.beta2 | -0.115 | 0.073 | -0.258 | 0.03 |
| sd.beta2 | 0.386 | 0.076 | 0.258 | 0.551 |
| mu.beta3 | -0.636 | 0.269 | -1.163 | -0.091 |
| sd.beta3 | 1.041 | 0.151 | 0.767 | 1.354 |
| mu.beta4 | -0.16 | 0.057 | -0.27 | -0.041 |
| sd.beta4 | 0.274 | 0.055 | 0.174 | 0.392 |
| mu.beta5 | -0.461 | 0.253 | -0.945 | 0.052 |
| sd.beta5 | 0.473 | 0.212 | 0.086 | 0.885 |
| mean.p | 0.376 | 0.047 | 0.282 | 0.467 |
| mu.lp | -0.511 | 0.205 | -0.936 | -0.134 |
| sd.lp | 1.285 | 0.185 | 0.959 | 1.684 |
| **Species-level parameters** | | |  |  |
| **Occupancy intercepts (logit scale)** | | |  |  |
| lpsi[1] | 0.308 | 0.43 | -0.427 | 1.301 |
| lpsi[2] | -5.312 | 1.169 | -7.781 | -3.233 |
| lpsi[3] | 1.938 | 0.725 | 0.763 | 3.566 |
| lpsi[4] | -4.879 | 1.167 | -7.268 | -2.661 |
| lpsi[5] | -2.497 | 1.096 | -4.29 | -0.038 |
| lpsi[6] | -1.981 | 0.558 | -2.975 | -0.754 |
| lpsi[7] | -2.773 | 0.627 | -3.99 | -1.459 |
| lpsi[8] | 3.006 | 0.738 | 1.861 | 4.585 |
| lpsi[9] | 2.389 | 0.659 | 1.382 | 3.984 |
| lpsi[12] | -1.099 | 0.418 | -1.851 | -0.214 |
| lpsi[14] | -3.432 | 1.211 | -5.503 | -0.658 |
| lpsi[10] | 1.081 | 0.324 | 0.48 | 1.765 |
| lpsi[15] | -2.644 | 1.209 | -4.594 | 0.134 |
| lpsi[16] | -2.578 | 1.354 | -4.617 | 0.721 |
| lpsi[17] | -3.148 | 0.857 | -4.729 | -1.325 |
| lpsi[18] | 2.161 | 0.801 | 0.919 | 4.055 |
| lpsi[20] | -3.254 | 1.155 | -5.411 | -0.826 |
| lpsi[11] | -1.97 | 1.161 | -3.899 | 0.883 |
| lpsi[19] | -2.131 | 0.902 | -3.722 | -0.096 |
| lpsi[21] | -1.31 | 0.748 | -2.556 | 0.376 |
| lpsi[22] | -3.308 | 0.835 | -4.876 | -1.541 |
| lpsi[23] | 0.823 | 0.605 | -0.133 | 2.263 |
| lpsi[49] | -3.284 | 1.425 | -6.095 | -0.28 |
| lpsi[24] | -3.956 | 1.77 | -7.35 | 0.141 |
| lpsi[25] | -0.267 | 0.337 | -0.912 | 0.401 |
| lpsi[26] | 0.703 | 0.423 | -0.043 | 1.599 |
| lpsi[27] | -1.109 | 1.209 | -3.007 | 1.658 |
| lpsi[28] | -2.115 | 0.822 | -3.511 | -0.267 |
| lpsi[13] | -2.693 | 0.651 | -3.957 | -1.359 |
| lpsi[29] | -1.679 | 1.111 | -3.488 | 0.773 |
| lpsi[30] | -1.225 | 0.286 | -1.782 | -0.673 |
| lpsi[31] | 1.642 | 0.35 | 0.999 | 2.361 |
| lpsi[32] | -0.378 | 0.698 | -1.581 | 1.163 |
| lpsi[33] | -0.106 | 1.301 | -2.154 | 2.778 |
| lpsi[34] | 0.423 | 1.432 | -1.462 | 4.563 |
| lpsi[35] | 0.626 | 0.487 | -0.223 | 1.699 |
| lpsi[50] | 0.479 | 0.413 | -0.231 | 1.336 |
| lpsi[36] | 0.407 | 0.398 | -0.316 | 1.264 |
| lpsi[37] | -0.933 | 0.532 | -1.879 | 0.227 |
| lpsi[38] | 0.876 | 0.991 | -0.832 | 3.025 |
| lpsi[39] | -2.642 | 0.802 | -4.063 | -0.825 |
| lpsi[40] | -1.297 | 1.167 | -3.201 | 1.411 |
| lpsi[41] | -1.303 | 1.797 | -3.852 | 3.159 |
| lpsi[42] | -2.879 | 1.44 | -5.38 | 0.368 |
| lpsi[43] | 2.095 | 1.011 | 0.507 | 4.537 |
| lpsi[44] | 0.686 | 0.338 | 0.069 | 1.419 |
| lpsi[45] | -0.68 | 0.42 | -1.436 | 0.204 |
| lpsi[46] | -3.964 | 1.475 | -6.576 | -0.569 |
| lpsi[47] | 1.506 | 0.707 | 0.396 | 3.19 |
| lpsi[51] | 2.316 | 0.627 | 1.243 | 3.68 |
| lpsi[52] | -2.543 | 0.569 | -3.598 | -1.385 |
| lpsi[48] | -0.455 | 0.309 | -1.029 | 0.189 |
| lpsi[53] | 0.148 | 1.18 | -1.608 | 3.066 |
| lpsi[54] | -1.694 | 1.068 | -3.651 | 0.55 |
| lpsi[55] | -0.767 | 0.362 | -1.445 | -0.012 |
| lpsi[56] | -1.945 | 1.464 | -4.123 | 1.662 |
| lpsi[57] | -1.035 | 0.917 | -2.615 | 1.08 |
| lpsi[58] | 1.121 | 0.7 | 0.135 | 2.763 |
| lpsi[59] | -0.861 | 1.596 | -2.783 | 3.739 |
| lpsi[60] | -0.403 | 0.857 | -1.638 | 1.81 |
| lpsi[61] | 4.701 | 0.879 | 3.212 | 6.664 |
| lpsi[62] | 1.508 | 0.405 | 0.787 | 2.385 |
| lpsi[63] | -3.088 | 0.892 | -4.722 | -1.212 |
| lpsi[64] | -3.909 | 1.211 | -6.073 | -1.182 |
| lpsi[65] | -1.212 | 0.386 | -1.944 | -0.447 |
| lpsi[66] | -0.09 | 0.755 | -1.32 | 1.646 |
| lpsi[67] | 0.011 | 0.627 | -0.905 | 1.56 |
| lpsi[68] | 2.206 | 0.445 | 1.404 | 3.152 |
| lpsi[69] | 2.436 | 0.516 | 1.517 | 3.554 |
| lpsi[70] | -1.354 | 0.319 | -1.993 | -0.724 |
| lpsi[71] | 2.585 | 0.699 | 1.52 | 4.239 |
| lpsi[72] | 0.63 | 0.813 | -0.745 | 2.433 |
| lpsi[73] | 0.026 | 0.321 | -0.571 | 0.701 |
| **Detection intercepts (logit scale)** | | |  |  |
| lp[1] | -0.024 | 0.267 | -0.546 | 0.496 |
| lp[2] | 0.285 | 1.164 | -1.951 | 2.564 |
| lp[3] | -0.419 | 0.215 | -0.816 | 0.024 |
| lp[4] | -0.201 | 1.297 | -2.814 | 2.333 |
| lp[5] | -1.432 | 0.804 | -2.987 | 0.072 |
| lp[6] | -0.039 | 0.649 | -1.367 | 1.169 |
| lp[7] | 0.284 | 0.82 | -1.372 | 1.888 |
| lp[8] | 0.514 | 0.156 | 0.21 | 0.821 |
| lp[9] | 0.398 | 0.191 | 0.038 | 0.771 |
| lp[12] | -0.239 | 0.337 | -0.943 | 0.389 |
| lp[14] | -0.892 | 1.435 | -3.785 | 1.685 |
| lp[10] | 0.65 | 0.22 | 0.225 | 1.086 |
| lp[15] | -1.332 | 1.263 | -3.696 | 1.045 |
| lp[16] | -1.275 | 1.368 | -4.07 | 1.235 |
| lp[17] | -0.298 | 1.09 | -2.601 | 1.757 |
| lp[18] | 0.269 | 0.175 | -0.055 | 0.641 |
| lp[20] | -1.857 | 0.912 | -3.598 | -0.069 |
| lp[11] | -1.894 | 0.895 | -3.644 | -0.135 |
| lp[19] | -1.266 | 0.77 | -2.807 | 0.124 |
| lp[21] | -1.178 | 0.544 | -2.233 | -0.121 |
| lp[22] | -0.184 | 0.914 | -2.086 | 1.487 |
| lp[23] | -0.393 | 0.252 | -0.867 | 0.117 |
| lp[49] | -2.133 | 0.969 | -4.033 | -0.321 |
| lp[24] | -1.258 | 1.602 | -4.581 | 1.662 |
| lp[25] | 0.179 | 0.288 | -0.377 | 0.77 |
| lp[26] | 0.481 | 0.18 | 0.131 | 0.848 |
| lp[27] | -1.652 | 0.93 | -3.288 | 0.142 |
| lp[28] | -1.315 | 0.615 | -2.436 | -0.066 |
| lp[13] | -0.326 | 0.704 | -1.679 | 1.082 |
| lp[29] | -1.558 | 0.962 | -3.387 | 0.151 |
| lp[30] | 1.463 | 0.261 | 0.972 | 1.992 |
| lp[31] | 1.347 | 0.176 | 1.014 | 1.707 |
| lp[32] | -1.469 | 0.364 | -2.123 | -0.703 |
| lp[33] | -2.042 | 0.804 | -3.303 | -0.358 |
| lp[34] | -1.916 | 0.426 | -2.708 | -1.004 |
| lp[35] | -0.328 | 0.306 | -0.907 | 0.264 |
| lp[50] | 0.387 | 0.194 | 0.023 | 0.771 |
| lp[36] | 0.164 | 0.207 | -0.231 | 0.576 |
| lp[37] | -0.741 | 0.287 | -1.286 | -0.153 |
| lp[38] | -1.568 | 0.4 | -2.195 | -0.634 |
| lp[39] | -0.702 | 0.88 | -2.497 | 0.97 |
| lp[40] | -1.828 | 0.816 | -3.244 | -0.173 |
| lp[41] | -2.37 | 1.094 | -4.254 | -0.27 |
| lp[42] | -1.894 | 1.267 | -4.352 | 0.448 |
| lp[43] | -0.936 | 0.199 | -1.296 | -0.503 |
| lp[44] | 0.474 | 0.241 | 0.003 | 0.95 |
| lp[45] | 0.014 | 0.284 | -0.57 | 0.599 |
| lp[46] | -1.143 | 1.539 | -4.218 | 1.722 |
| lp[47] | -0.518 | 0.243 | -0.966 | -0.039 |
| lp[51] | 0.802 | 0.157 | 0.485 | 1.118 |
| lp[52] | 0.326 | 0.791 | -1.26 | 1.873 |
| lp[48] | 1.101 | 0.205 | 0.71 | 1.535 |
| lp[53] | -1.821 | 0.508 | -2.742 | -0.768 |
| lp[54] | -2.162 | 0.659 | -3.304 | -0.711 |
| lp[55] | 0.213 | 0.329 | -0.43 | 0.852 |
| lp[56] | -2.022 | 1.166 | -4.188 | 0.178 |
| lp[57] | -1.59 | 0.63 | -2.741 | -0.338 |
| lp[58] | -0.16 | 0.243 | -0.634 | 0.294 |
| lp[59] | -1.415 | 1.031 | -3.212 | 0.395 |
| lp[60] | -1.064 | 0.389 | -1.804 | -0.304 |
| lp[61] | 1.819 | 0.161 | 1.522 | 2.152 |
| lp[62] | 0.627 | 0.19 | 0.267 | 1.004 |
| lp[63] | -0.892 | 0.783 | -2.462 | 0.591 |
| lp[64] | -0.969 | 1.404 | -3.663 | 1.854 |
| lp[65] | 0.314 | 0.205 | -0.069 | 0.724 |
| lp[66] | -1.14 | 0.449 | -1.95 | -0.21 |
| lp[67] | -0.491 | 0.404 | -1.344 | 0.223 |
| lp[68] | 1.473 | 0.185 | 1.129 | 1.853 |
| lp[69] | 0.986 | 0.165 | 0.667 | 1.32 |
| lp[70] | 0.67 | 0.274 | 0.135 | 1.21 |
| lp[71] | 0.659 | 0.159 | 0.352 | 0.987 |
| lp[72] | -1.802 | 0.337 | -2.4 | -1.09 |
| lp[73] | 0.685 | 0.297 | 0.092 | 1.27 |
| **Site-level results** | |  |  |  |
| **Species richness at each site** | | |  |  |
| Nsite[1] | 26.228 | 3.028 | 21 | 32 |
| Nsite[2] | 35.687 | 2.548 | 31 | 41 |
| Nsite[3] | 35.889 | 2.48 | 31 | 41 |
| Nsite[4] | 30.552 | 3.059 | 25 | 37 |
| Nsite[5] | 32.523 | 2.612 | 28 | 38 |
| Nsite[6] | 28.784 | 3.127 | 23 | 35 |
| Nsite[7] | 29.798 | 2.76 | 25 | 36 |
| Nsite[8] | 26.477 | 3.132 | 20 | 33 |
| Nsite[9] | 28.675 | 3.188 | 22 | 35 |
| Nsite[10] | 26.123 | 2.526 | 22 | 31 |
| Nsite[11] | 28.596 | 3.165 | 23 | 35 |
| Nsite[12] | 29.818 | 2.624 | 25 | 35 |
| Nsite[13] | 35.329 | 2.408 | 31 | 40 |
| Nsite[14] | 26.062 | 2.885 | 21 | 32 |
| Nsite[15] | 25.364 | 2.449 | 21 | 30 |
| Nsite[16] | 31.303 | 2.497 | 27 | 36 |
| Nsite[17] | 29.591 | 2.852 | 24 | 35 |
| Nsite[18] | 28.11 | 3.175 | 22 | 34 |
| Nsite[19] | 32.936 | 2.339 | 29 | 38 |
| Nsite[20] | 29.137 | 2.585 | 25 | 34 |
| Nsite[21] | 32.588 | 2.654 | 28 | 38 |
| Nsite[22] | 30.719 | 2.622 | 26 | 36 |
| Nsite[23] | 24.543 | 2.484 | 20 | 30 |
| Nsite[24] | 27.382 | 3.089 | 22 | 34 |
| Nsite[25] | 35.865 | 2.338 | 32 | 41 |
| Nsite[26] | 27.085 | 3.058 | 21 | 33 |
| Nsite[27] | 22.694 | 3.305 | 16 | 29 |
| Nsite[28] | 31.337 | 2.928 | 26 | 37 |
| Nsite[29] | 28.775 | 2.595 | 24 | 34 |
| Nsite[30] | 26.555 | 3.287 | 20 | 33 |
| Nsite[31] | 29.324 | 2.831 | 24 | 35 |
| Nsite[32] | 28.945 | 2.912 | 24 | 35 |
| Nsite[33] | 32.624 | 2.75 | 28 | 38 |
| Nsite[34] | 26.729 | 2.696 | 22 | 32 |
| Nsite[35] | 28.416 | 2.992 | 23 | 34 |
| Nsite[36] | 26.403 | 3.254 | 20 | 33 |
| Nsite[37] | 23.556 | 3.263 | 18 | 30 |
| Nsite[38] | 24.925 | 3.274 | 19 | 32 |
| Nsite[39] | 27.345 | 2.833 | 22 | 33 |
| Nsite[40] | 27.547 | 3.022 | 22 | 34 |
| Nsite[41] | 27.093 | 3.167 | 21 | 34 |
| Nsite[42] | 28.687 | 3.071 | 23 | 35 |
| Nsite[43] | 28.61 | 2.827 | 23 | 34.025 |
| Nsite[44] | 31.033 | 2.87 | 26 | 37 |
| Nsite[45] | 26.597 | 2.548 | 22 | 32 |
| Nsite[46] | 32.795 | 2.604 | 28 | 38 |
| Nsite[47] | 23.566 | 2.97 | 18 | 30 |
| Nsite[48] | 29.192 | 2.876 | 24 | 35 |
| Nsite[49] | 24.648 | 3.311 | 18 | 31 |
| Nsite[50] | 26.477 | 2.779 | 21.975 | 32 |
| Nsite[51] | 33.847 | 2.76 | 29 | 39.025 |
| Nsite[52] | 29.941 | 2.609 | 25 | 35.025 |
| Nsite[53] | 27.129 | 3.233 | 21 | 34 |
| Nsite[54] | 24.33 | 3.332 | 18 | 31 |
| Nsite[55] | 30.796 | 2.331 | 27 | 36 |
| Nsite[56] | 30.743 | 2.866 | 25 | 36 |
| Nsite[57] | 31.337 | 2.821 | 26 | 37 |
| Nsite[58] | 32.911 | 2.895 | 28 | 39 |
| Nsite[59] | 33.749 | 2.487 | 29 | 39 |
| Nsite[60] | 24.249 | 2.934 | 19 | 30 |
| Nsite[61] | 30.817 | 2.312 | 27 | 36 |
| Nsite[62] | 27.778 | 2.905 | 22 | 34 |
| Nsite[63] | 18.362 | 2.763 | 13 | 24 |
| Nsite[64] | 33.21 | 2.511 | 29 | 38 |
| Nsite[65] | 31.901 | 2.454 | 27 | 37 |
| Nsite[66] | 25.167 | 2.967 | 20 | 31 |
| Nsite[67] | 28.759 | 3.2 | 23 | 35 |
| Nsite[68] | 27.607 | 3.34 | 21 | 34 |
| Nsite[69] | 31.004 | 2.606 | 26 | 36 |
| Nsite[70] | 33.462 | 2.621 | 29 | 39 |
| Nsite[71] | 29.321 | 2.731 | 24 | 35 |
| Nsite[72] | 28.596 | 2.938 | 23 | 35 |
| Nsite[73] | 27.742 | 2.944 | 22 | 34 |
| Nsite[74] | 31.99 | 3.053 | 26 | 38 |
| Nsite[75] | 32.911 | 2.397 | 28 | 38 |
| Nsite[76] | 26.776 | 2.742 | 22 | 32 |
| Nsite[77] | 27.016 | 2.556 | 22 | 32 |
| Nsite[78] | 26.643 | 2.597 | 22 | 32 |
| Nsite[79] | 28.158 | 2.929 | 23 | 34 |
| Nsite[80] | 26.35 | 3.059 | 21 | 32 |
| Nsite[81] | 26.671 | 2.228 | 23 | 31 |
| Nsite[82] | 27.83 | 2.481 | 23 | 33 |
| Nsite[83] | 28.283 | 2.875 | 23 | 34 |
| Nsite[84] | 25.834 | 3.046 | 20 | 32 |
| Nsite[85] | 25.68 | 3.312 | 19.975 | 33 |
| Nsite[86] | 24.872 | 2.744 | 20 | 30 |
| Nsite[87] | 27.002 | 2.793 | 22 | 33 |
| Nsite[88] | 31.403 | 2.841 | 26 | 37 |
| Nsite[89] | 27.208 | 2.731 | 22 | 33 |
| Nsite[90] | 25.694 | 3.098 | 20 | 32 |
| Nsite[91] | 30.677 | 3.116 | 25 | 37 |
| Nsite[92] | 31.433 | 3.057 | 26 | 38 |
| Nsite[93] | 27.826 | 2.954 | 23 | 34 |
| Nsite[94] | 29.456 | 2.239 | 25 | 34 |
| Nsite[95] | 27.793 | 3.21 | 22 | 34 |
| Nsite[96] | 33.248 | 2.53 | 29 | 38 |
| Nsite[97] | 28.387 | 3.033 | 23 | 35 |
| Nsite[98] | 31.625 | 1.977 | 28 | 36 |
| Nsite[99] | 29.446 | 2.56 | 25 | 35 |
| Nsite[100] | 27.975 | 2.852 | 23 | 33.025 |
| Nsite[101] | 31.271 | 2.893 | 26 | 37 |
| Nsite[102] | 30.025 | 3.167 | 24 | 36 |
| Nsite[103] | 29.733 | 3.383 | 23 | 36 |
| Nsite[104] | 33.997 | 2.999 | 29 | 40 |
| Nsite[105] | 33.533 | 3.025 | 28 | 40 |
| Nsite[106] | 33.123 | 3.05 | 28 | 39 |
| Nsite[107] | 24.03 | 2.323 | 20 | 29 |
| Nsite[108] | 25.696 | 2.181 | 22 | 30 |
| Nsite[109] | 24.621 | 2.843 | 19 | 30 |
| Nsite[110] | 33.045 | 3.141 | 27 | 39 |
| Nsite[111] | 31.34 | 2.366 | 27 | 36 |
| Nsite[112] | 28.5 | 3.456 | 22 | 35 |
| Nsite[113] | 26.257 | 3.629 | 19 | 33 |
| Nsite[114] | 30.123 | 2.634 | 25 | 36 |
| Nsite[115] | 25.185 | 2.69 | 20 | 31 |
| Nsite[116] | 37.506 | 2.504 | 33 | 43 |
| Nsite[117] | 29.23 | 1.987 | 26 | 33 |
| Nsite[118] | 31.567 | 3.275 | 25 | 38 |
| Nsite[119] | 31.11 | 3.264 | 25 | 38 |
| Nsite[120] | 24.289 | 3.32 | 18 | 31 |
| Nsite[121] | 27.233 | 2.707 | 22 | 33 |
| Nsite[122] | 28.522 | 2.14 | 25 | 33 |
| Nsite[123] | 24.59 | 3.548 | 18 | 31 |
| Nsite[124] | 26.556 | 3.052 | 21 | 33 |
| Nsite[125] | 26.863 | 3.483 | 20 | 34 |
| Nsite[126] | 26.344 | 3.322 | 20 | 33 |
| Nsite[127] | 23.112 | 2.747 | 18 | 29 |
| Nsite[128] | 27.206 | 2.429 | 23 | 32 |
| Nsite[129] | 30.836 | 2.263 | 27 | 36 |
| Nsite[130] | 29.682 | 3.217 | 24 | 36 |
| Nsite[131] | 26.694 | 3.093 | 21 | 33 |
| Nsite[132] | 24.618 | 3.154 | 19 | 31 |
| Nsite[133] | 25.155 | 3.344 | 19 | 32 |
| Nsite[134] | 27.446 | 3.226 | 21 | 34 |
| Nsite[135] | 30.09 | 2.545 | 25 | 35 |
| Nsite[136] | 31.463 | 2.919 | 26 | 38 |
| Nsite[137] | 22.647 | 3.08 | 17 | 29 |
| Nsite[138] | 23.247 | 2.795 | 18 | 29 |
| Nsite[139] | 22.496 | 2.901 | 17 | 28 |
| Nsite[140] | 32.487 | 2.284 | 28 | 37 |
| Nsite[141] | 27.44 | 3.429 | 21 | 34 |
| Nsite[142] | 27.201 | 3.54 | 21 | 34 |
| Nsite[143] | 29.474 | 2.927 | 24 | 35 |
| Nsite[144] | 22.949 | 2.962 | 18 | 29 |
| Nsite[145] | 29.104 | 2.834 | 24 | 35 |
| Nsite[146] | 26.575 | 2.824 | 21 | 32 |
| Nsite[147] | 28.626 | 2.408 | 25 | 34 |
| Nsite[148] | 29.943 | 2.665 | 25 | 36 |
| Nsite[149] | 31.519 | 2.24 | 28 | 36 |
| Nsite[150] | 30.267 | 2.408 | 26 | 35 |
| Nsite[151] | 27.482 | 2.812 | 22 | 33 |
| Nsite[152] | 27.981 | 3.385 | 22 | 35 |
| Nsite[153] | 27.284 | 3.308 | 21 | 34 |
| Nsite[154] | 27.212 | 3.39 | 21 | 34 |
| Nsite[155] | 31.241 | 2.842 | 26 | 37 |
| Nsite[156] | 29.547 | 2.829 | 24 | 35 |
| Nsite[157] | 26.564 | 2.46 | 22 | 32 |
| Nsite[158] | 28.664 | 3.314 | 22 | 35 |
| Nsite[159] | 28.469 | 2.284 | 24 | 33 |
| Nsite[160] | 26.787 | 2.97 | 21 | 33 |
| Nsite[161] | 32.035 | 2.864 | 27 | 38 |
| Nsite[162] | 27.528 | 2.951 | 22 | 34 |
| Nsite[163] | 26.204 | 3.494 | 20 | 33 |
| Nsite[164] | 24.511 | 3.07 | 19 | 31 |
| Nsite[165] | 29.962 | 3.102 | 24 | 36 |
| Nsite[166] | 28.242 | 2.74 | 23 | 34 |
| Nsite[167] | 28.062 | 3.308 | 22 | 35 |
| Nsite[168] | 33.753 | 2.518 | 29 | 39 |
| Nsite[169] | 31.566 | 2.793 | 26 | 37 |
| Nsite[170] | 30.828 | 2.553 | 26 | 36 |
| Nsite[171] | 24.675 | 2.803 | 19 | 30 |
| Nsite[172] | 26.137 | 2.645 | 21 | 32 |
| Nsite[173] | 26.442 | 2.585 | 22 | 32 |
| Nsite[174] | 24.888 | 2.66 | 20 | 30 |
| Nsite[175] | 26.88 | 2.903 | 22 | 33 |
| Nsite[176] | 26.39 | 2.653 | 22 | 32 |
| Nsite[177] | 27.762 | 2.758 | 23 | 33 |
| Nsite[178] | 30.534 | 2.902 | 25 | 37 |
| Nsite[179] | 30.381 | 2.374 | 26 | 35 |
| Nsite[180] | 28.174 | 3.236 | 22 | 35 |
| Nsite[181] | 25.48 | 3.259 | 19 | 32 |
| Nsite[182] | 26.388 | 3.251 | 20 | 33 |
| Nsite[183] | 34.099 | 2.785 | 29 | 40 |
| Nsite[184] | 25.565 | 2.861 | 20 | 31 |
| Nsite[185] | 36.419 | 2.277 | 32 | 41 |
| Nsite[186] | 33.985 | 2.712 | 29 | 40 |
| Nsite[187] | 25.874 | 3.204 | 20 | 32 |
| Nsite[188] | 30.59 | 3.144 | 24 | 37 |
| Nsite[189] | 30.56 | 3.096 | 25 | 37 |
| Nsite[190] | 33.869 | 3.054 | 28 | 40 |
| Nsite[191] | 27.9 | 3.097 | 22 | 34 |
| Nsite[192] | 28.012 | 3.296 | 22 | 35 |
| Nsite[193] | 25.391 | 3.407 | 19 | 32 |
| Nsite[194] | 22.112 | 3.21 | 16 | 28 |
| Nsite[195] | 24.37 | 2.938 | 19 | 30 |
| Nsite[196] | 23.665 | 2.846 | 18 | 29 |
| Nsite[197] | 18.147 | 2.74 | 13 | 24 |
| Nsite[198] | 29.652 | 3.171 | 24 | 36 |
| Nsite[199] | 29.571 | 2.556 | 25 | 35 |
| Nsite[200] | 27.548 | 3.087 | 22 | 34 |
| Nsite[201] | 26.855 | 2.905 | 21 | 33 |
| Nsite[202] | 27.728 | 2.976 | 22 | 34 |
| Nsite[203] | 23.755 | 2.537 | 19 | 29 |
| Nsite[204] | 25.311 | 2.351 | 21 | 30 |
| Nsite[205] | 34.78 | 2.483 | 30 | 40 |
| Nsite[206] | 31.578 | 2.503 | 27 | 37 |
| Nsite[207] | 33.248 | 2.359 | 29 | 38 |
| Nsite[208] | 29.683 | 2.991 | 24 | 36 |
| Nsite[209] | 26.157 | 2.695 | 21 | 32 |
| Nsite[210] | 22.514 | 2.964 | 17 | 29 |
| Nsite[211] | 20.768 | 2.803 | 15 | 26 |
| Nsite[212] | 21.102 | 2.574 | 16 | 26 |
| Nsite[213] | 19.352 | 2.584 | 15 | 25 |
| Nsite[214] | 18.867 | 2.72 | 14 | 24 |
| Nsite[215] | 31.339 | 2.955 | 26 | 37 |
| Nsite[216] | 21.393 | 3.016 | 16 | 28 |
| Nsite[217] | 21.382 | 3.036 | 16 | 27 |
| Nsite[218] | 26.441 | 2.806 | 21 | 32 |
| Nsite[219] | 19.868 | 2.663 | 15 | 25 |
| Nsite[220] | 21.265 | 2.712 | 16 | 27 |
| Nsite[221] | 28.524 | 3.173 | 23 | 35 |
| Nsite[222] | 19.668 | 2.749 | 15 | 25 |
| Nsite[223] | 21.706 | 2.963 | 16 | 28 |
| Nsite[224] | 19.686 | 2.677 | 15 | 25 |
| Nsite[225] | 21.718 | 3.04 | 16 | 28 |
| Nsite[226] | 18.071 | 2.789 | 13 | 24 |
| Nsite[227] | 28.351 | 3.22 | 22 | 35 |
| Nsite[228] | 30.195 | 3.075 | 24 | 37 |
| Nsite[229] | 17.751 | 2.905 | 12 | 24 |
| Nsite[230] | 17.041 | 2.537 | 12 | 22 |
| Nsite[231] | 31.101 | 2.92 | 26 | 37 |
| Nsite[232] | 27.742 | 3.167 | 22 | 34 |
| Nsite[233] | 29.227 | 3.064 | 23 | 35 |
| Nsite[234] | 29.215 | 2.954 | 24 | 35 |
| Nsite[235] | 22.176 | 2.788 | 17 | 28 |
| Nsite[236] | 28.108 | 2.906 | 23 | 34 |
| Nsite[237] | 28.632 | 3.013 | 23 | 35 |
| Nsite[238] | 26.961 | 3.134 | 21 | 33 |
| Nsite[239] | 23.883 | 2.975 | 18 | 30 |
| Nsite[240] | 20.961 | 3.126 | 15 | 27 |
| Nsite[241] | 19.82 | 3.183 | 14 | 26 |
| Nsite[242] | 19.981 | 2.999 | 14 | 26 |
| Nsite[243] | 24.675 | 3.08 | 19 | 31 |
| Nsite[244] | 20.791 | 3.261 | 15 | 27 |
| Nsite[245] | 22.05 | 3.102 | 16 | 28 |
| Nsite[246] | 20.408 | 3.142 | 14 | 27 |

**Table S3.** Output of community occupancy model for aggregating the basic surveys (1 km transects) for the aggregation scheme AS3x3. Results include the estimates for community-level parameters, including the covariates for occupancy (beta1: linear effect of forest cover; beta2: quadratic effect of forest cover; beta3: linear effects of temperature; beta4: quadratic effect of temperature; beta5: linear effect of precipitation). Species-level parameters include occupancy (lpsi) and detectability (lp) whose identity for the 73 species is numbered according to Table S1. Site level results include the estimate of community size for the 185 samples sites (Nsite).

|  | mean | sd | 2.50% | 97.50% |
| --- | --- | --- | --- | --- |
| **Community-level**  **parameters** | | |  |  |
| mean.psi | 0.369 | 0.066 | 0.249 | 0.508 |
| mu.lpsi | -0.546 | 0.288 | -1.104 | 0.031 |
| sd.lpsi | 2.136 | 0.233 | 1.733 | 2.64 |
| mu.beta1 | 0.036 | 0.124 | -0.198 | 0.281 |
| sd.beta1 | 0.754 | 0.114 | 0.549 | 1.004 |
| mu.beta2 | -0.073 | 0.054 | -0.178 | 0.032 |
| sd.beta2 | 0.125 | 0.076 | 0.004 | 0.275 |
| mu.beta3 | -0.717 | 0.31 | -1.313 | -0.11 |
| sd.beta3 | 1.042 | 0.171 | 0.727 | 1.412 |
| mu.beta4 | -0.149 | 0.062 | -0.273 | -0.024 |
| sd.beta4 | 0.258 | 0.063 | 0.137 | 0.387 |
| mu.beta5 | -0.498 | 0.296 | -1.063 | 0.084 |
| sd.beta5 | 0.531 | 0.238 | 0.022 | 0.973 |
| mean.p | 0.321 | 0.039 | 0.245 | 0.402 |
| mu.lp | -0.753 | 0.182 | -1.128 | -0.397 |
| sd.lp | 1.28 | 0.157 | 1.012 | 1.628 |
| **Species-level parameters** | | |  |  |
| **Occupancy intercepts (logit scale)** | | |  |  |
| lpsi[1] | 1.091 | 0.611 | 0.172 | 2.617 |
| lpsi[2] | -4.724 | 1.053 | -6.842 | -2.717 |
| lpsi[3] | 2.483 | 0.987 | 0.984 | 4.642 |
| lpsi[4] | -4.143 | 1.115 | -6.285 | -1.882 |
| lpsi[5] | -2.168 | 0.807 | -3.652 | -0.4 |
| lpsi[6] | -1.619 | 0.564 | -2.624 | -0.404 |
| lpsi[7] | -1.905 | 0.641 | -3.085 | -0.549 |
| lpsi[8] | 2.441 | 0.515 | 1.573 | 3.629 |
| lpsi[9] | 1.434 | 0.421 | 0.753 | 2.382 |
| lpsi[12] | -1.395 | 0.334 | -2.068 | -0.749 |
| lpsi[14] | -3.424 | 1.016 | -5.373 | -1.293 |
| lpsi[10] | 0.9 | 0.299 | 0.343 | 1.536 |
| lpsi[15] | -2.159 | 1.146 | -4.016 | 0.504 |
| lpsi[16] | -2.43 | 1.302 | -4.381 | 0.842 |
| lpsi[17] | -2.339 | 0.966 | -3.986 | -0.199 |
| lpsi[18] | 2.943 | 0.959 | 1.39 | 5.067 |
| lpsi[20] | -2.012 | 2.009 | -4.716 | 4.212 |
| lpsi[11] | -1.285 | 1.298 | -3.271 | 1.826 |
| lpsi[19] | -1.583 | 0.963 | -3.07 | 0.693 |
| lpsi[21] | -1.984 | 0.486 | -2.918 | -1.028 |
| lpsi[22] | -3.282 | 0.729 | -4.775 | -1.867 |
| lpsi[23] | 0.831 | 0.443 | 0.132 | 1.853 |
| lpsi[49] | -2.477 | 1.385 | -4.897 | 0.392 |
| lpsi[24] | -3.652 | 1.393 | -6.159 | -0.37 |
| lpsi[25] | 0.07 | 0.345 | -0.565 | 0.786 |
| lpsi[26] | 1.212 | 0.38 | 0.518 | 2.005 |
| lpsi[27] | -1.084 | 1.258 | -2.891 | 1.946 |
| lpsi[28] | -1.968 | 0.74 | -3.351 | -0.373 |
| lpsi[13] | -2.392 | 0.655 | -3.636 | -1.035 |
| lpsi[29] | -1.962 | 0.879 | -3.341 | 0.323 |
| lpsi[30] | -0.79 | 0.265 | -1.324 | -0.283 |
| lpsi[31] | 1.923 | 0.379 | 1.242 | 2.738 |
| lpsi[32] | -0.178 | 0.684 | -1.372 | 1.323 |
| lpsi[33] | -1.1 | 0.979 | -2.483 | 1.569 |
| lpsi[34] | -0.318 | 0.81 | -1.627 | 1.574 |
| lpsi[35] | 0.724 | 0.468 | -0.04 | 1.773 |
| lpsi[50] | 0.679 | 0.375 | 0.015 | 1.467 |
| lpsi[36] | 0.903 | 0.387 | 0.23 | 1.75 |
| lpsi[37] | -0.527 | 0.448 | -1.369 | 0.421 |
| lpsi[38] | 1.307 | 1.027 | -0.437 | 3.54 |
| lpsi[39] | -2.853 | 0.724 | -4.217 | -1.335 |
| lpsi[40] | -1.756 | 0.951 | -3.275 | 0.567 |
| lpsi[41] | -0.882 | 1.585 | -3.188 | 2.898 |
| lpsi[42] | -3.099 | 1.555 | -5.502 | 1.057 |
| lpsi[43] | 0.978 | 0.628 | 0.011 | 2.435 |
| lpsi[44] | 0.523 | 0.292 | -0.015 | 1.134 |
| lpsi[45] | -0.602 | 0.318 | -1.195 | 0.019 |
| lpsi[46] | -3.207 | 1.424 | -5.598 | 0.06 |
| lpsi[47] | 0.928 | 0.467 | 0.181 | 1.986 |
| lpsi[51] | 2.439 | 0.664 | 1.359 | 3.909 |
| lpsi[52] | -1.824 | 0.705 | -3.044 | -0.234 |
| lpsi[48] | 0.309 | 0.311 | -0.287 | 0.934 |
| lpsi[53] | 1.597 | 1.429 | -0.865 | 4.626 |
| lpsi[54] | -1.327 | 0.93 | -3.028 | 0.656 |
| lpsi[55] | -0.64 | 0.363 | -1.313 | 0.145 |
| lpsi[56] | -0.923 | 1.535 | -3.218 | 2.82 |
| lpsi[57] | -1.255 | 0.727 | -2.479 | 0.397 |
| lpsi[58] | 0.772 | 0.352 | 0.143 | 1.548 |
| lpsi[59] | -0.892 | 1.415 | -2.471 | 3.073 |
| lpsi[60] | -0.189 | 0.589 | -1.105 | 1.246 |
| lpsi[61] | 4.297 | 0.792 | 2.992 | 5.989 |
| lpsi[62] | 1.294 | 0.365 | 0.65 | 2.087 |
| lpsi[63] | -3.05 | 0.711 | -4.426 | -1.596 |
| lpsi[64] | -3.619 | 1.29 | -5.848 | -0.534 |
| lpsi[65] | -0.708 | 0.339 | -1.361 | -0.04 |
| lpsi[66] | -0.181 | 0.61 | -1.189 | 1.158 |
| lpsi[67] | 0.47 | 0.608 | -0.513 | 1.881 |
| lpsi[68] | 2.247 | 0.376 | 1.575 | 3.044 |
| lpsi[69] | 2.509 | 0.46 | 1.696 | 3.496 |
| lpsi[70] | -1.044 | 0.291 | -1.614 | -0.471 |
| lpsi[71] | 2.642 | 0.705 | 1.586 | 4.423 |
| lpsi[72] | 0.329 | 0.771 | -1.014 | 2.004 |
| lpsi[73] | 0.144 | 0.319 | -0.458 | 0.796 |
| **Detection intercepts (logit scale)** | | |  |  |
| lp[1] | -0.511 | 0.206 | -0.908 | -0.1 |
| lp[2] | -0.396 | 1.021 | -2.411 | 1.562 |
| lp[3] | -0.701 | 0.166 | -1.014 | -0.356 |
| lp[4] | -1.492 | 1.031 | -3.487 | 0.544 |
| lp[5] | -1.485 | 0.671 | -2.836 | -0.226 |
| lp[6] | -0.955 | 0.475 | -1.935 | -0.09 |
| lp[7] | -1.169 | 0.585 | -2.366 | -0.072 |
| lp[8] | 0.502 | 0.142 | 0.232 | 0.787 |
| lp[9] | 0.524 | 0.169 | 0.187 | 0.846 |
| lp[12] | 0.036 | 0.264 | -0.496 | 0.54 |
| lp[14] | -0.953 | 1.38 | -3.574 | 1.815 |
| lp[10] | 0.434 | 0.173 | 0.097 | 0.781 |
| lp[15] | -1.958 | 0.99 | -3.929 | -0.141 |
| lp[16] | -1.808 | 1.13 | -4.149 | 0.191 |
| lp[17] | -1.573 | 0.893 | -3.393 | 0.016 |
| lp[18] | 0.125 | 0.134 | -0.126 | 0.404 |
| lp[20] | -2.623 | 0.963 | -4.585 | -0.908 |
| lp[11] | -2.49 | 0.731 | -3.851 | -1.05 |
| lp[19] | -1.637 | 0.754 | -3.111 | -0.253 |
| lp[21] | -0.59 | 0.43 | -1.477 | 0.208 |
| lp[22] | -0.582 | 0.706 | -2.076 | 0.698 |
| lp[23] | -0.316 | 0.217 | -0.766 | 0.082 |
| lp[49] | -2.548 | 0.896 | -4.245 | -0.792 |
| lp[24] | -1.893 | 1.352 | -4.484 | 0.72 |
| lp[25] | -0.209 | 0.226 | -0.665 | 0.234 |
| lp[26] | 0.389 | 0.15 | 0.105 | 0.682 |
| lp[27] | -1.823 | 0.89 | -3.407 | -0.206 |
| lp[28] | -1.344 | 0.54 | -2.404 | -0.304 |
| lp[13] | -0.814 | 0.593 | -1.98 | 0.358 |
| lp[29] | -1.413 | 0.764 | -3.131 | -0.101 |
| lp[30] | 1.348 | 0.222 | 0.922 | 1.8 |
| lp[31] | 1.07 | 0.148 | 0.782 | 1.356 |
| lp[32] | -1.558 | 0.32 | -2.154 | -0.908 |
| lp[33] | -1.572 | 0.688 | -3.018 | -0.355 |
| lp[34] | -1.649 | 0.428 | -2.421 | -0.803 |
| lp[35] | -0.546 | 0.246 | -1.032 | -0.081 |
| lp[50] | 0.325 | 0.162 | 0.007 | 0.649 |
| lp[36] | -0.066 | 0.181 | -0.416 | 0.283 |
| lp[37] | -0.865 | 0.231 | -1.299 | -0.404 |
| lp[38] | -1.735 | 0.294 | -2.241 | -1.068 |
| lp[39] | -0.76 | 0.748 | -2.35 | 0.627 |
| lp[40] | -1.437 | 0.828 | -3.058 | 0.06 |
| lp[41] | -2.733 | 0.863 | -4.251 | -1.038 |
| lp[42] | -1.9 | 1.303 | -4.511 | 0.523 |
| lp[43] | -0.746 | 0.213 | -1.162 | -0.326 |
| lp[44] | 0.487 | 0.195 | 0.109 | 0.875 |
| lp[45] | 0.083 | 0.241 | -0.386 | 0.554 |
| lp[46] | -2.132 | 1.225 | -4.649 | 0.134 |
| lp[47] | -0.451 | 0.21 | -0.883 | -0.043 |
| lp[51] | 0.771 | 0.141 | 0.507 | 1.053 |
| lp[52] | -1.007 | 0.658 | -2.302 | 0.238 |
| lp[48] | 0.743 | 0.17 | 0.412 | 1.075 |
| lp[53] | -2.402 | 0.393 | -3.04 | -1.501 |
| lp[54] | -2.432 | 0.489 | -3.356 | -1.421 |
| lp[55] | -0.286 | 0.239 | -0.768 | 0.171 |
| lp[56] | -2.872 | 0.845 | -4.395 | -1.193 |
| lp[57] | -1.618 | 0.513 | -2.61 | -0.616 |
| lp[58] | -0.031 | 0.185 | -0.397 | 0.315 |
| lp[59] | -1.404 | 0.847 | -3.126 | -0.011 |
| lp[60] | -0.957 | 0.335 | -1.635 | -0.334 |
| lp[61] | 1.821 | 0.164 | 1.512 | 2.155 |
| lp[62] | 0.477 | 0.154 | 0.171 | 0.786 |
| lp[63] | -0.803 | 0.644 | -2.099 | 0.403 |
| lp[64] | -1.311 | 1.453 | -4.038 | 1.546 |
| lp[65] | 0.23 | 0.176 | -0.105 | 0.576 |
| lp[66] | -1.225 | 0.361 | -1.895 | -0.509 |
| lp[67] | -0.938 | 0.309 | -1.521 | -0.325 |
| lp[68] | 1.357 | 0.158 | 1.057 | 1.673 |
| lp[69] | 0.865 | 0.141 | 0.589 | 1.147 |
| lp[70] | 0.636 | 0.23 | 0.193 | 1.09 |
| lp[71] | 0.619 | 0.141 | 0.347 | 0.89 |
| lp[72] | -1.795 | 0.301 | -2.346 | -1.172 |
| lp[73] | 0.086 | 0.215 | -0.331 | 0.5 |
| **Site-level results** | |  |  |  |
| **Species richness at each site** | | |  |  |
| Nsite[1] | 28.456 | 3.043 | 23 | 35 |
| Nsite[2] | 38.941 | 2.131 | 35 | 44 |
| Nsite[3] | 33.085 | 2.933 | 28 | 39 |
| Nsite[4] | 34.715 | 2.307 | 31 | 39.025 |
| Nsite[5] | 28.803 | 3.016 | 23 | 35 |
| Nsite[6] | 32.94 | 2.922 | 27 | 39 |
| Nsite[7] | 33.991 | 2.979 | 28 | 40 |
| Nsite[8] | 29.846 | 2.469 | 25 | 35 |
| Nsite[9] | 35.334 | 2.209 | 31 | 40 |
| Nsite[10] | 31.182 | 3.154 | 25 | 37 |
| Nsite[11] | 27.395 | 2.745 | 22 | 33 |
| Nsite[12] | 27.742 | 2.732 | 23 | 33 |
| Nsite[13] | 32.463 | 2.583 | 28 | 38 |
| Nsite[14] | 30.547 | 2.703 | 26 | 36 |
| Nsite[15] | 34.927 | 2.474 | 30 | 40 |
| Nsite[16] | 30.677 | 2.32 | 27 | 36 |
| Nsite[17] | 33.053 | 2.48 | 28 | 38 |
| Nsite[18] | 30.071 | 2.941 | 25 | 36 |
| Nsite[19] | 32.646 | 2.941 | 27 | 39 |
| Nsite[20] | 32.608 | 2.67 | 28 | 38 |
| Nsite[21] | 28.75 | 3.009 | 23 | 35 |
| Nsite[22] | 23.447 | 2.721 | 18 | 29 |
| Nsite[23] | 32.627 | 2.837 | 28 | 38.025 |
| Nsite[24] | 33.872 | 3.067 | 28 | 40 |
| Nsite[25] | 29.668 | 2.441 | 25 | 35 |
| Nsite[26] | 30.693 | 2.807 | 25 | 36 |
| Nsite[27] | 34.419 | 2.549 | 30 | 40 |
| Nsite[28] | 27.743 | 2.254 | 24 | 32 |
| Nsite[29] | 26.061 | 3.206 | 20 | 32 |
| Nsite[30] | 29.937 | 2.711 | 25 | 35 |
| Nsite[31] | 30.957 | 2.621 | 26 | 36 |
| Nsite[32] | 32.592 | 2.312 | 28 | 37 |
| Nsite[33] | 33.881 | 2.469 | 30 | 39 |
| Nsite[34] | 26.092 | 2.707 | 21 | 31 |
| Nsite[35] | 30.447 | 2.872 | 25 | 36 |
| Nsite[36] | 26.1 | 3.523 | 19 | 33 |
| Nsite[37] | 27.582 | 2.891 | 22 | 33.025 |
| Nsite[38] | 35.728 | 2.23 | 32 | 40 |
| Nsite[39] | 32.525 | 2.938 | 27 | 39 |
| Nsite[40] | 29.884 | 2.715 | 25 | 35 |
| Nsite[41] | 24.953 | 3.011 | 19 | 31 |
| Nsite[42] | 26.066 | 3.427 | 20 | 33 |
| Nsite[43] | 37.338 | 2.112 | 34 | 42 |
| Nsite[44] | 33.521 | 2.493 | 29 | 38 |
| Nsite[45] | 31.031 | 2.341 | 27 | 36 |
| Nsite[46] | 24.836 | 2.935 | 19 | 31 |
| Nsite[47] | 32.507 | 2.976 | 27 | 38 |
| Nsite[48] | 23.358 | 3.271 | 17 | 30 |
| Nsite[49] | 30.573 | 2.646 | 26 | 36 |
| Nsite[50] | 32.81 | 2.823 | 27.975 | 39 |
| Nsite[51] | 34.82 | 2.083 | 31 | 39 |
| Nsite[52] | 34.812 | 2.786 | 29 | 40 |
| Nsite[53] | 27.837 | 2.449 | 24 | 33 |
| Nsite[54] | 29.513 | 2.302 | 25 | 34 |
| Nsite[55] | 28.092 | 2.31 | 24 | 33 |
| Nsite[56] | 30.291 | 3.043 | 25 | 37 |
| Nsite[57] | 28.05 | 2.979 | 23 | 34 |
| Nsite[58] | 29.328 | 2.081 | 26 | 34 |
| Nsite[59] | 30.806 | 3.071 | 25 | 37 |
| Nsite[60] | 28.884 | 2.664 | 24 | 34 |
| Nsite[61] | 26.166 | 2.98 | 20 | 32 |
| Nsite[62] | 27.734 | 2.966 | 22 | 34 |
| Nsite[63] | 31.989 | 3.096 | 26 | 38 |
| Nsite[64] | 32.04 | 2.554 | 27 | 37 |
| Nsite[65] | 29.623 | 2.469 | 25 | 35 |
| Nsite[66] | 33.426 | 2.964 | 28 | 39.025 |
| Nsite[67] | 30.782 | 2.264 | 27 | 35 |
| Nsite[68] | 31.361 | 2.783 | 26 | 37 |
| Nsite[69] | 34.549 | 2.514 | 30 | 40 |
| Nsite[70] | 33.497 | 2.114 | 30 | 38 |
| Nsite[71] | 31.872 | 2.333 | 28 | 37 |
| Nsite[72] | 32.947 | 2.787 | 28 | 39 |
| Nsite[73] | 30.815 | 3.091 | 25 | 37 |
| Nsite[74] | 36.594 | 2.632 | 32 | 42 |
| Nsite[75] | 33.095 | 3.032 | 27.975 | 39 |
| Nsite[76] | 25.261 | 2.37 | 21 | 30 |
| Nsite[77] | 26.135 | 2.203 | 22 | 31 |
| Nsite[78] | 29.074 | 3.11 | 23 | 35 |
| Nsite[79] | 27.599 | 3.167 | 21 | 34 |
| Nsite[80] | 32.986 | 3.2 | 27 | 39 |
| Nsite[81] | 27.404 | 3.266 | 21 | 34 |
| Nsite[82] | 34.356 | 2.495 | 30 | 39 |
| Nsite[83] | 29.541 | 2.41 | 25 | 34 |
| Nsite[84] | 37.681 | 2.367 | 33 | 43 |
| Nsite[85] | 27.494 | 3.42 | 21 | 34 |
| Nsite[86] | 31.997 | 1.677 | 29 | 36 |
| Nsite[87] | 26.951 | 3.591 | 20 | 34 |
| Nsite[88] | 28 | 3.1 | 22 | 34 |
| Nsite[89] | 29.05 | 3.455 | 22 | 36 |
| Nsite[90] | 31.204 | 2.474 | 27 | 36 |
| Nsite[91] | 27.477 | 3.116 | 22 | 34 |
| Nsite[92] | 26.625 | 2.923 | 21 | 33 |
| Nsite[93] | 27.668 | 2.572 | 23 | 33 |
| Nsite[94] | 32.601 | 2.481 | 28 | 37 |
| Nsite[95] | 30.076 | 2.666 | 25 | 36 |
| Nsite[96] | 27.205 | 3.161 | 21 | 33 |
| Nsite[97] | 29.383 | 3.43 | 23 | 36 |
| Nsite[98] | 28.923 | 3.426 | 22 | 36 |
| Nsite[99] | 28.685 | 3.143 | 23 | 35 |
| Nsite[100] | 27.825 | 3.371 | 21 | 34 |
| Nsite[101] | 32.356 | 2.814 | 27 | 38 |
| Nsite[102] | 30.614 | 3.354 | 24 | 37 |
| Nsite[103] | 31.903 | 2.197 | 28 | 37 |
| Nsite[104] | 32.408 | 2.533 | 28 | 38 |
| Nsite[105] | 31.874 | 2.545 | 27 | 37 |
| Nsite[106] | 27.622 | 2.62 | 23 | 33 |
| Nsite[107] | 23.955 | 2.619 | 19 | 29 |
| Nsite[108] | 33.433 | 2.331 | 29 | 38 |
| Nsite[109] | 27.802 | 3.197 | 22 | 34 |
| Nsite[110] | 30.081 | 2.93 | 25 | 36 |
| Nsite[111] | 28.544 | 3.341 | 22 | 35 |
| Nsite[112] | 34.074 | 3.105 | 28 | 40 |
| Nsite[113] | 28.85 | 3.091 | 23 | 35 |
| Nsite[114] | 31.769 | 2.902 | 26 | 38 |
| Nsite[115] | 26.409 | 3.04 | 21 | 32 |
| Nsite[116] | 29.078 | 2.359 | 25 | 34 |
| Nsite[117] | 27.467 | 2.751 | 23 | 33 |
| Nsite[118] | 30.267 | 2.44 | 26 | 35 |
| Nsite[119] | 29.767 | 2.524 | 25 | 35 |
| Nsite[120] | 28.535 | 3.1 | 23 | 35 |
| Nsite[121] | 35.062 | 2.12 | 31 | 40 |
| Nsite[122] | 28.38 | 2.803 | 23 | 34 |
| Nsite[123] | 25.811 | 3.149 | 20 | 32 |
| Nsite[124] | 31.443 | 2.862 | 26 | 37 |
| Nsite[125] | 30.27 | 2.926 | 25 | 36 |
| Nsite[126] | 28.959 | 3.326 | 23 | 36 |
| Nsite[127] | 32.518 | 2.885 | 27 | 38 |
| Nsite[128] | 31.97 | 3.097 | 26 | 38 |
| Nsite[129] | 30.534 | 3.033 | 25 | 37 |
| Nsite[130] | 26.916 | 2.527 | 22 | 32 |
| Nsite[131] | 28.889 | 2.297 | 25 | 33 |
| Nsite[132] | 30.197 | 3.066 | 24 | 36 |
| Nsite[133] | 29.222 | 2.356 | 25 | 34 |
| Nsite[134] | 33.923 | 2.122 | 30 | 38 |
| Nsite[135] | 30.184 | 3.424 | 24 | 37 |
| Nsite[136] | 29.832 | 3.254 | 24 | 36 |
| Nsite[137] | 35.364 | 2.762 | 30 | 41 |
| Nsite[138] | 26.983 | 3.08 | 21 | 33 |
| Nsite[139] | 26.943 | 2.838 | 22 | 33 |
| Nsite[140] | 32.892 | 3.15 | 27 | 39 |
| Nsite[141] | 32.689 | 2.791 | 27 | 38 |
| Nsite[142] | 35.505 | 2.959 | 30 | 41 |
| Nsite[143] | 32.251 | 3.166 | 26 | 39 |
| Nsite[144] | 35.224 | 2.518 | 30 | 40 |
| Nsite[145] | 30.427 | 3.267 | 24 | 37 |
| Nsite[146] | 26.319 | 3.26 | 20 | 33 |
| Nsite[147] | 29.796 | 2.946 | 24 | 36 |
| Nsite[148] | 32.262 | 2.966 | 27 | 38 |
| Nsite[149] | 25.651 | 2.998 | 20 | 32 |
| Nsite[150] | 24.838 | 2.269 | 21 | 29 |
| Nsite[151] | 24.876 | 2.966 | 19 | 31 |
| Nsite[152] | 19.108 | 2.902 | 14 | 25 |
| Nsite[153] | 31.39 | 2.969 | 26 | 38 |
| Nsite[154] | 36.3 | 1.891 | 33 | 40 |
| Nsite[155] | 33.862 | 2.199 | 30 | 38 |
| Nsite[156] | 29.834 | 2.002 | 26 | 34 |
| Nsite[157] | 23.845 | 3.057 | 18 | 30 |
| Nsite[158] | 21.863 | 2.972 | 16 | 28 |
| Nsite[159] | 22.055 | 2.77 | 17 | 28 |
| Nsite[160] | 20.562 | 2.517 | 16 | 26 |
| Nsite[161] | 32.738 | 3.078 | 27 | 39 |
| Nsite[162] | 20.804 | 2.87 | 15.975 | 27 |
| Nsite[163] | 19.532 | 2.792 | 14 | 25 |
| Nsite[164] | 22.731 | 3.126 | 17 | 29 |
| Nsite[165] | 23.222 | 3.201 | 17 | 30 |
| Nsite[166] | 26.895 | 2.774 | 22 | 32 |
| Nsite[167] | 20.131 | 2.947 | 15 | 26 |
| Nsite[168] | 20.723 | 2.826 | 15 | 27 |
| Nsite[169] | 22.298 | 2.784 | 17 | 28 |
| Nsite[170] | 29.724 | 2.743 | 25 | 35 |
| Nsite[171] | 18.657 | 2.807 | 13 | 24 |
| Nsite[172] | 21.395 | 2.965 | 16 | 27 |
| Nsite[173] | 17.933 | 2.689 | 13 | 23 |
| Nsite[174] | 34.072 | 2.646 | 29 | 40 |
| Nsite[175] | 29.354 | 3.142 | 23 | 36 |
| Nsite[176] | 30.444 | 2.737 | 25 | 36 |
| Nsite[177] | 30.055 | 3.022 | 24 | 36 |
| Nsite[178] | 28.848 | 3.155 | 23 | 35 |
| Nsite[179] | 21.372 | 2.485 | 17 | 27 |
| Nsite[180] | 20.476 | 3.196 | 14 | 27 |
| Nsite[181] | 26.862 | 3.218 | 21 | 33 |
| Nsite[182] | 21.483 | 3.036 | 16 | 28 |
| Nsite[183] | 24.381 | 3.351 | 18 | 31 |
| Nsite[184] | 21.78 | 3.237 | 15 | 28 |
| Nsite[185] | 23.453 | 3.107 | 18 | 30 |

**Table S4.** Intercepts of the probability of occupancy (*psi*) and detectability (*p*) for each of the observed 73 species under the two aggregation schemes AS2x2 and AS3x3.

| **Number of species** | **Scientific name** | **Common name** | **Representative** | **AS2x2** | | **AS3x3** | |
| --- | --- | --- | --- | --- | --- | --- | --- |
|  |  |  | **avian family** | ***psi*** | ***p*** | ***psi*** | ***p*** |
| 1 | *Aegithalos caudatus* | Long-tailed Tit | - | 0.576 | 0.494 | 0.749 | 0.375 |
| 2 | *Alauda arvensis* | Eurasian Skylark | - | 0.005 | 0.571 | 0.009 | 0.402 |
| 3 | *Alectoris rufa* | Red-legged Partridge | - | 0.874 | 0.397 | 0.923 | 0.332 |
| 4 | *Anthus campestris* | Tawny Pipit | - | 0.008 | 0.450 | 0.016 | 0.184 |
| 5 | *Athene noctua* | Little Owl | - | 0.076 | 0.193 | 0.103 | 0.185 |
| 6 | *Calandrella brachydactyla* | Greater Short-toed Lark | - | 0.121 | 0.490 | 0.165 | 0.278 |
| 7 | *Calandrella rufescens* | Lesser Short-toed Lark | - | 0.059 | 0.571 | 0.130 | 0.237 |
| 8 | *Carduelis cannabina* | Common Linnet | Fringillidae | 0.953 | 0.626 | 0.920 | 0.623 |
| 9 | *Carduelis carduelis* | European Goldfinch | Fringillidae | 0.916 | 0.598 | 0.807 | 0.628 |
| 10 | *Certhia brachydactyla* | Short-toed Treecreeper | - | 0.250 | 0.441 | 0.199 | 0.509 |
| 11 | *Cettia cetti* | Cetti's Warbler | | 0.031 | 0.291 | 0.032 | 0.278 |
| 12 | *Chloris chloris* | European Greenfinch | Fringillidae | 0.747 | 0.657 | 0.711 | 0.607 |
| 13 | *Clamator glandarius* | Great Spotted Cuckoo | - | 0.066 | 0.209 | 0.104 | 0.124 |
| 14 | *Columba livia* | Rock Dove | - | 0.071 | 0.218 | 0.081 | 0.141 |
| 15 | *Columba oenas* | Stock Dove | - | 0.041 | 0.426 | 0.088 | 0.172 |
| 16 | *Columba palumbus* | Common Wood Pigeon | - | 0.897 | 0.567 | 0.950 | 0.531 |
| 17 | *Coracias garrulus* | European Roller | - | 0.037 | 0.135 | 0.118 | 0.068 |
| 18 | *Corvus corax* | Northern Raven | - | 0.122 | 0.131 | 0.217 | 0.077 |
| 19 | *Corvus corone* | Carrion Crow | - | 0.106 | 0.220 | 0.170 | 0.163 |
| 20 | *Corvus monedula* | Western Jackdaw | - | 0.212 | 0.235 | 0.121 | 0.357 |
| 21 | *Coturnix coturnix* | Common Quail | - | 0.035 | 0.454 | 0.036 | 0.358 |
| 22 | *Cuculus canorus* | Common Cuckoo | - | 0.695 | 0.403 | 0.697 | 0.422 |
| 23 | *Cyanistes caeruleus* | Blue Tit | Paridae | 0.036 | 0.106 | 0.077 | 0.073 |
| 24 | *Dendrocopos major* | Great Spotted Woodpecker | - | 0.019 | 0.221 | 0.025 | 0.131 |
| 25 | *Emberiza calandra* | Corn Bunting | - | 0.434 | 0.545 | 0.518 | 0.448 |
| 26 | *Emberiza cia* | Rock Bunting | - | 0.669 | 0.618 | 0.771 | 0.596 |
| 27 | *Emberiza cirlus* | Cirl Bunting | - | 0.248 | 0.161 | 0.253 | 0.139 |
| 28 | *Erithacus rubecula* | European Robin | Turdidae | 0.108 | 0.212 | 0.123 | 0.207 |
| 29 | *Erythropygia galactotes* | Rufous-tailed Scrub Robin | Turdidae | 0.063 | 0.419 | 0.084 | 0.307 |
| 30 | *Ficedula hypoleuca* | European Pied Flycatcher | - | 0.157 | 0.174 | 0.123 | 0.196 |
| 31 | *Fringilla coelebs* | Common Chaffinch | Fringillidae | 0.227 | 0.812 | 0.312 | 0.794 |
| 32 | *Galerida cristata/theklae* | Crested/Thekla Lark | - | 0.838 | 0.794 | 0.872 | 0.745 |
| 33 | *Garrulus glandarius* | Eurasian Jay | - | 0.407 | 0.187 | 0.456 | 0.174 |
| 34 | *Hippolais polyglotta* | Melodious Warbler | Sylviidae | 0.474 | 0.115 | 0.250 | 0.172 |
| 35 | *Lanius meridionalis* | Southern Grey Shrike | - | 0.604 | 0.128 | 0.421 | 0.161 |
| 36 | *Lanius senator* | Woodchat Shrike | - | 0.652 | 0.419 | 0.673 | 0.367 |
| 37 | *Lophophanes cristatus* | European Crested Tit | Paridae | 0.618 | 0.595 | 0.664 | 0.581 |
| 38 | *Loxia curvirostra* | Red Crossbill | Fringillidae | 0.600 | 0.541 | 0.712 | 0.483 |
| 39 | *Lullula arborea* | Woodlark | - | 0.282 | 0.323 | 0.371 | 0.296 |
| 40 | *Luscinia megarhynchos* | Common Nightingale | Turdidae | 0.706 | 0.173 | 0.787 | 0.150 |
| 41 | *Melanocorypha calandra* | Calandra Lark | - | 0.066 | 0.331 | 0.055 | 0.319 |
| 42 | *Monticola solitarius* | Blue Rock Thrush | Turdidae | 0.215 | 0.138 | 0.147 | 0.192 |
| 43 | *Motacilla alba* | White Wagtail | - | 0.214 | 0.086 | 0.293 | 0.061 |
| 44 | *Motacilla cinerea* | Grey Wagtail | - | 0.053 | 0.131 | 0.043 | 0.130 |
| 45 | *Muscicapa striata* | Spotted Flycatcher | - | 0.890 | 0.282 | 0.727 | 0.322 |
| 46 | *Oenanthe hispanica* | Black-eared Wheatear | Turdidae | 0.665 | 0.616 | 0.628 | 0.620 |
| 47 | *Oenanthe leucura* | Black Wheatear | Turdidae | 0.336 | 0.503 | 0.354 | 0.521 |
| 48 | *Oenanthe oenanthe* | Northern Wheatear | Turdidae | 0.019 | 0.242 | 0.039 | 0.106 |
| 49 | *Oriolus oriolus* | Eurasian Golden Oriole | - | 0.818 | 0.373 | 0.717 | 0.389 |
| 50 | *Parus major* | Great Tit | Paridae | 0.910 | 0.690 | 0.920 | 0.684 |
| 51 | *Passer domesticus* | House Sparrow | - | 0.073 | 0.581 | 0.139 | 0.268 |
| 52 | *Periparus ater* | Coal Tit | Paridae | 0.388 | 0.750 | 0.577 | 0.678 |
| 53 | *Petronia petronia* | Rock Sparrow | - | 0.537 | 0.139 | 0.832 | 0.083 |
| 54 | *Phoenicurus ochruros* | Black Redstart | Turdidae | 0.155 | 0.103 | 0.210 | 0.081 |
| 55 | *Phylloscopus bonelli* | Western Bonelli's Warbler | Sylviidae | 0.317 | 0.553 | 0.345 | 0.429 |
| 56 | *Phylloscopus collybita* | Common Chiffchaff | Sylviidae | 0.125 | 0.117 | 0.284 | 0.054 |
| 57 | *Pica pica* | Common Magpie | - | 0.262 | 0.169 | 0.222 | 0.166 |
| 58 | *Picus sharpei* | Iberian Green Woodpecker | - | 0.754 | 0.460 | 0.684 | 0.492 |
| 59 | *Pyrrhocorax pyrrhocorax* | Red-billed Chough | - | 0.297 | 0.195 | 0.291 | 0.197 |
| 60 | *Saxicola torquatus* | Common Stonechat | Turdidae | 0.401 | 0.257 | 0.453 | 0.277 |
| 61 | *Serinus serinus* | European Serin | Fringillidae | 0.991 | 0.860 | 0.987 | 0.861 |
| 62 | *Streptopelia turtur* | European Turtle Dove | - | 0.819 | 0.652 | 0.785 | 0.617 |
| 63 | *Sturnus unicolor* | Spotless Starling | - | 0.044 | 0.291 | 0.045 | 0.309 |
| 64 | *Sylvia borin* | Garden Warbler | Sylviidae | 0.020 | 0.275 | 0.026 | 0.212 |
| 65 | *Sylvia cantillans* | Subalpine Warbler | Sylviidae | 0.229 | 0.578 | 0.330 | 0.557 |
| 66 | *Sylvia conspicillata* | Spectacled Warbler | Sylviidae | 0.478 | 0.242 | 0.455 | 0.227 |
| 67 | *Sylvia hortensis* | Western Orphean Warbler | Sylviidae | 0.503 | 0.380 | 0.615 | 0.281 |
| 68 | *Sylvia melanocephala* | Sardinian Warbler | Sylviidae | 0.901 | 0.814 | 0.904 | 0.795 |
| 69 | *Sylvia undata* | Dartford Warbler | Sylviidae | 0.920 | 0.728 | 0.925 | 0.704 |
| 70 | *Troglodytes troglodytes* | Winter Wren | - | 0.205 | 0.662 | 0.260 | 0.654 |
| 71 | *Turdus merula* | Common Blackbird | Turdidae | 0.930 | 0.659 | 0.934 | 0.650 |
| 72 | *Turdus viscivorus* | Mistle Thrush | Turdidae | 0.652 | 0.142 | 0.581 | 0.142 |
| 73 | *Upupa epops* | Hoopoe | - | 0.507 | 0.665 | 0.536 | 0.521 |

**Figure S1.** Number of detected species considering the spatial transects conducted for each site (represented by lines with different colours) in both aggregation schemes of different grid cell size: a) AS2x2 (with 246 sites from 1 to 5 transects); b) AS3x3 (with 185 sites from 1 to 7 transects).


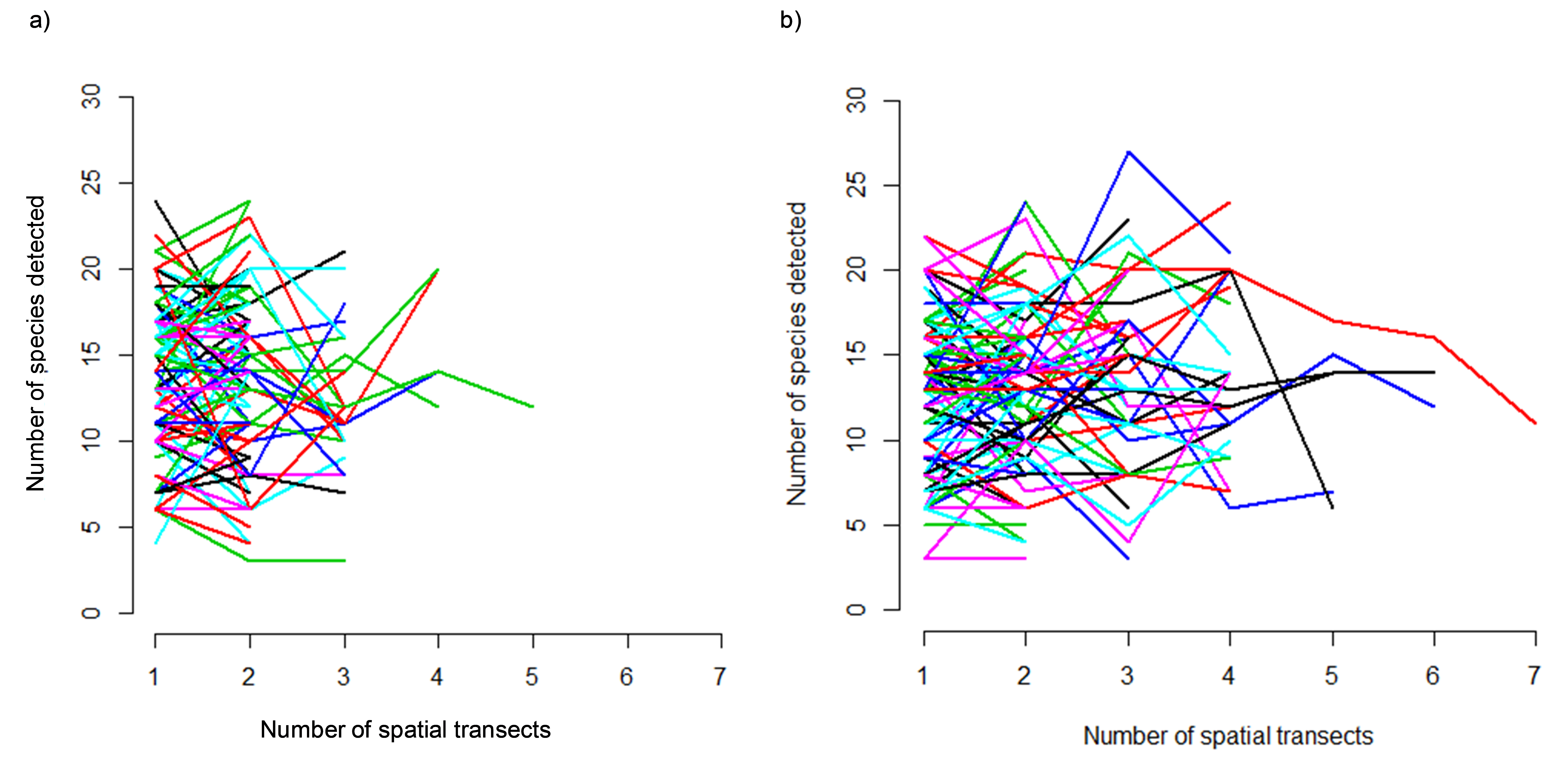


**Figure S2.** Observed species richness per cell site considering both aggregation schemes a) AS2x2 and b) AS3x3 in the classical bird survey (*n*=246 and 185 cell sites for each aggregation scheme). Red line shows a mean of approximately 15 and 17 species for each aggregation scheme, respectively.


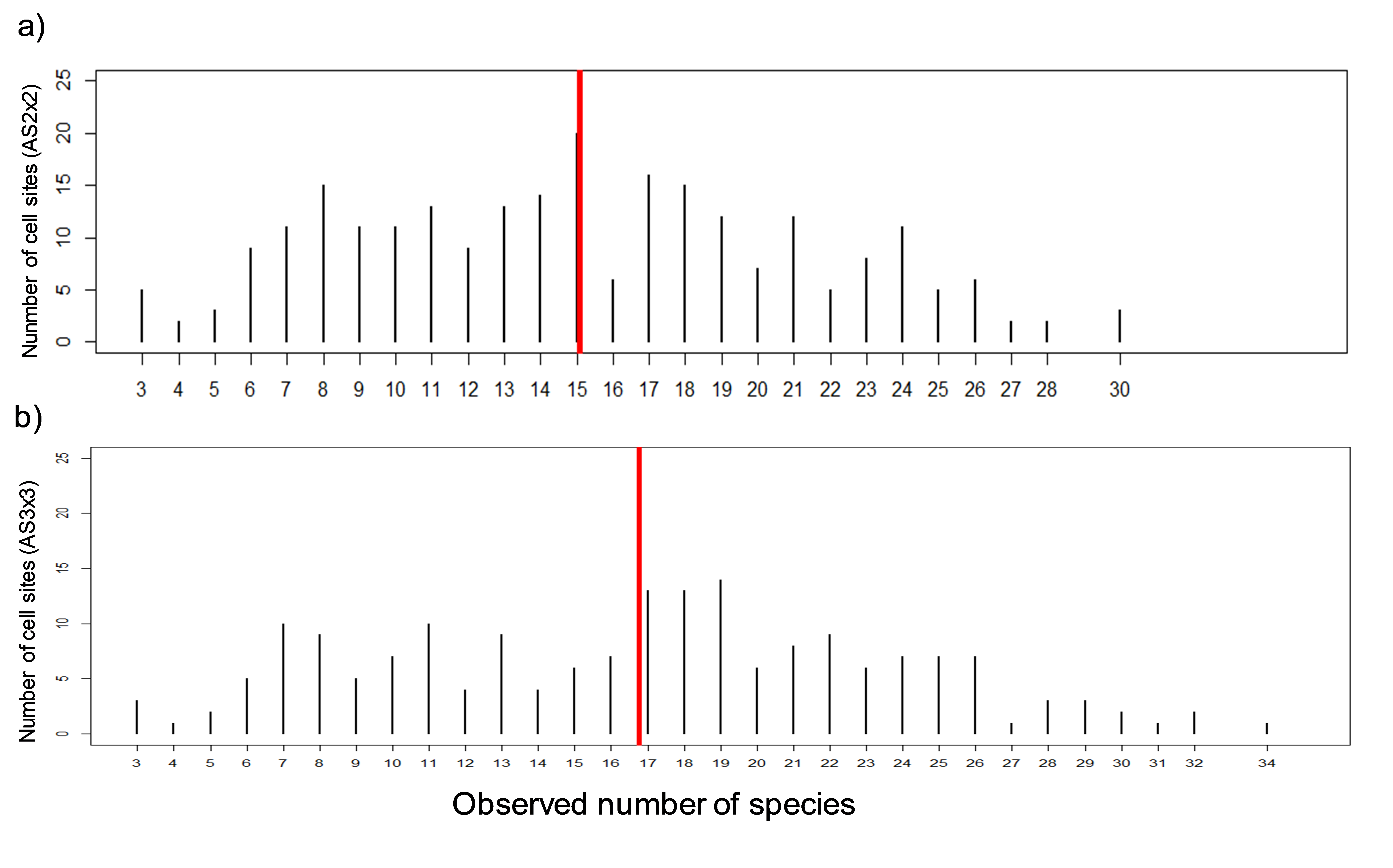


**Figure S3.** Mean of estimates of detection probability and occupancy probability for the historical bird transect in the region of Murcia (with 95% CI). a) transects grouped in 246 sites for the aggregation scheme AS2x2. b) transects grouped in 185 sites for the aggregation scheme AS3x3. Colours represent the most representative avian families: black: other avian families; red: Sylviidae; green: Turdidae; blue light: Fringillidae; blue: Paridae.


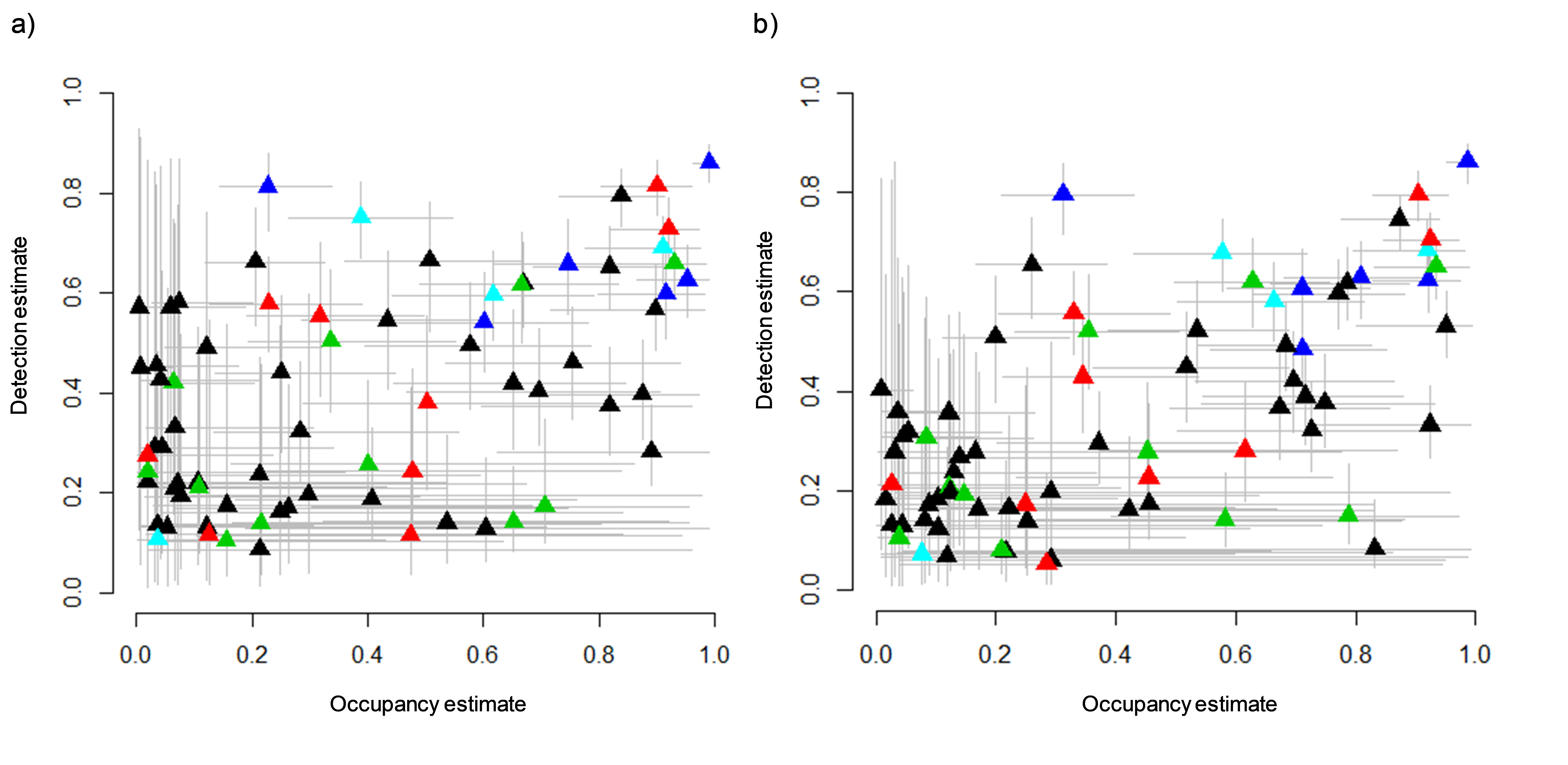


**Figure S4.** Species-specific predictions of occupancy probability as a function of forest cover, temperature and precipitation at grid cells of each aggregation scheme: a-c) AS2x2 and d-f) AS3x3, under a community occupancy model. Each line represents one of the 73 observed species for each aggregation scheme. Note that one covariate is kept at the observed average for the computation of the prediction for the other covariate and vice versa.


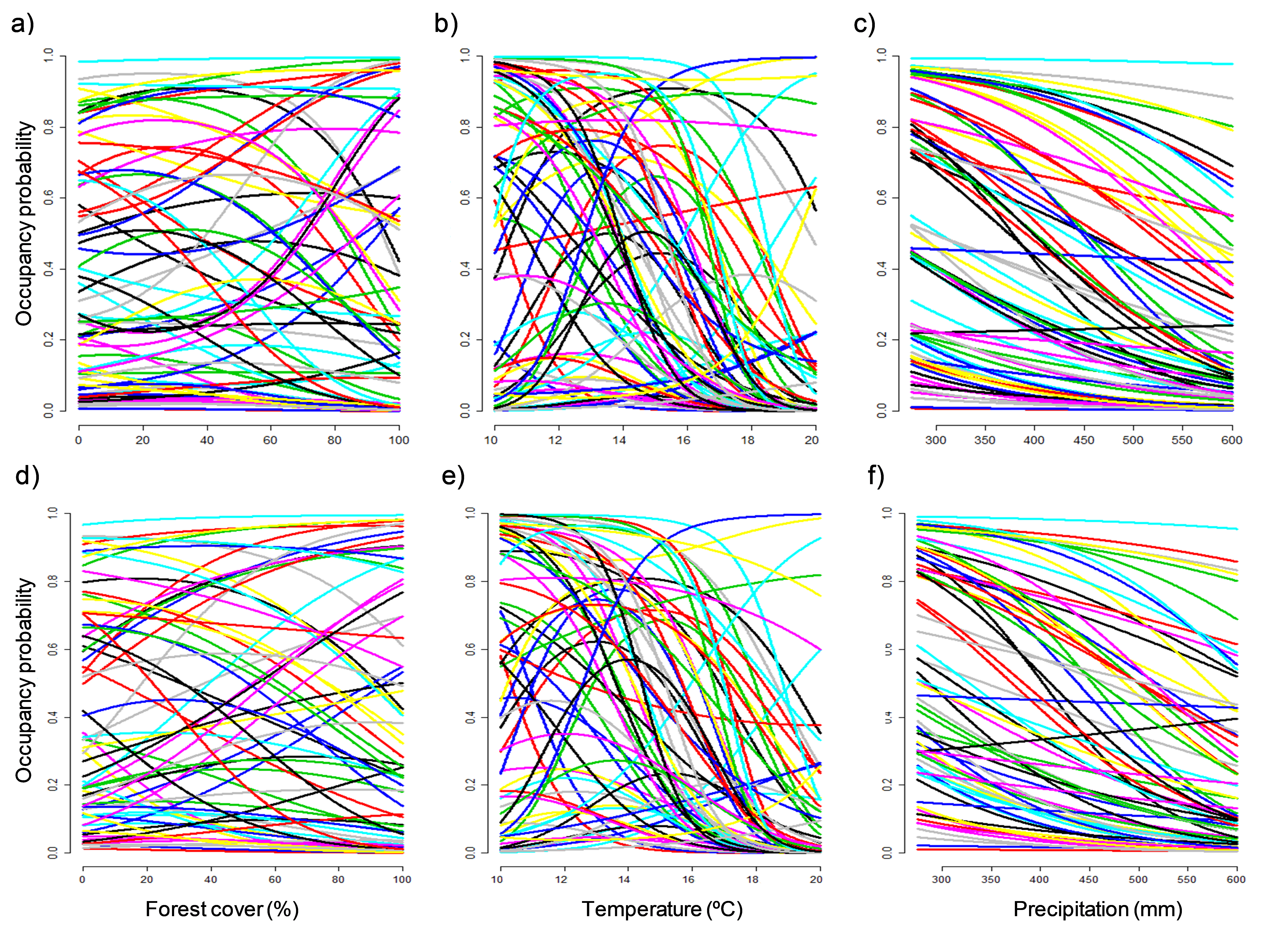

Supplement: Supplementary file 2 [file ECE3-9-825-s002.doc]
